# Toward large reasoning models: A survey of reinforced reasoning with large language models

**DOI:** 10.1016/j.patter.2025.101370

**Published:** 2025-10-10

**Authors:** Fengli Xu, Qianyue Hao, Chenyang Shao, Zefang Zong, Yu Li, Jingwei Wang, Yunke Zhang, Jingyi Wang, Xiaochong Lan, Jiahui Gong, Tianjian Ouyang, Fanjin Meng, Yuwei Yan, Qinglong Yang, Yiwen Song, Sijian Ren, Xinyuan Hu, Jie Feng, Chen Gao, Yong Li

**Affiliations:** 1 Tsinghua University, Beijing, China; 2 HKUST (GZ), Guangzhou, China; 3 Emory University, Atlanta, GA, USA

**Keywords:** large language model, LLM, LLM reasoning, reinforcement learning, neural scaling law

## Abstract

Language has long been an essential tool for human reasoning. The rise of large language models (LLMs) has led to research on their application in complex reasoning tasks. Researchers are exploring the concept of “thought,” which represents intermediate reasoning steps, allowing LLMs to emulate humanlike reasoning processes. Recent work has applied reinforcement learning (RL) to train LLMs by searching for high-quality reasoning trajectories through trial-and-error exploration. In parallel, studies also demonstrate that allowing LLMs to “think” with longer chains of intermediate tokens at test time can also substantially improve reasoning accuracy. The combination of training and test-time advancements outlines a path toward large reasoning models. This survey reviews recent progress in LLM reasoning. It covers foundational concepts behind LLMs and the key technical components that contribute to the development of large reasoning models, and it highlights popular open-source projects for building these models. The survey concludes by discussing ongoing challenges and future research directions in this field.

## The bigger picture

Reasoning—the ability to think through problems step-by-step, integrate diverse knowledge, and adapt to new situations—is central to human intelligence. Large language models (LLMs) have recently shown surprising progress in this area, shifting from simply predicting the next word to executing multi-step, structured thought processes. This transformation is driven by two complementary forces. First, at test time, providing models with more “thinking time” and guiding them to explore multiple reasoning paths can substantially boost accuracy. Second, increasingly sophisticated reinforcement learning-based post-training techniques are expanding the upper limits of models’ intrinsic reasoning capabilities.

This survey synthesizes the rapidly expanding body of research into a coherent framework for what we term “large reasoning models” (LRMs). We explain how automated construction of reasoning data, process-level reward models, and test-time search strategies are pushing the frontier of AI reasoning. These developments matter not only for benchmark performance but also for real-world applications where reasoning quality directly impacts safety, reliability, and scientific discovery, such as medical diagnosis, engineering design, legal analysis, and educational tools. By comparing proprietary breakthroughs like OpenAI’s o1 series with emerging open-source replications, we illuminate the technical pathways and trade-offs in building LRMs. Our road map reveals that scaling reasoning ability is not solely about increasing model size. It also depends on how models are trained, how they are guided at test time, and how feedback loops between the two can be optimized. Ultimately, advancing LRMs may bring AI nearer to robust general problem-solving, offering systems that can reason transparently, adapt to complex challenges, and move us closer to the arrival of artificial general intelligence (AGI).

## Teaser

This survey reviews recent advances in enhancing the reasoning capabilities of large language models (LLMs) through reinforcement learning, automated data construction, and test-time scaling. An emerging paradigm of “large reasoning models” is introduced in this review, highlighting milestones such as OpenAI’s o1 series and analyzing open-source efforts to replicate advanced reasoning. By unifying train-time and test-time scaling, the authors provide a taxonomy, comparative analysis, and road map for developing LLMs with humanlike reasoning in complex, multi-step problem-solving.

## Introduction

If there is a severe deficit of language, there will be severe deficit of thought.—Noam Chomsky

Fueled by advances in deep learning and the availability of web-scale datasets, large language models (LLMs) have emerged as a transformative paradigm on the path toward artificial general intelligence (AGI). These massive AI models typically adopt transformer architecture and are pretrained on large-scale text corpus with the next-token prediction task.^1^ The neural scaling law demonstrates that their performance improves significantly as the model size and training data increase.^2^ More importantly, LLMs also unlock remarkable *emergent abilities* that are absent in smaller models,^3^ such as in-context learning,^4^ role playing,^5^ and analogical reasoning.^6^ These abilities allow LLMs to go beyond natural language processing problems to facilitate a wider range of tasks, such as code generation,^7^ robotic control,^8^ and autonomous agents.^9^

Among these abilities, humanlike reasoning has garnered significant attention from both academia and industry, since it demonstrates great potential for LLMs to generalize to complex real-world problems through abstract and logical reasoning. A notable breakthrough in this area is the “chain-of-thought” prompting technique,^10^ which can elicit step-by-step humanlike reasoning processes at test time without any additional training. Such intuitive prompting techniques have been proven effective to substantially improve the reasoning accuracy of pretrained LLMs, which has also led to the development of more advanced prompting techniques like tree-of-thoughts (ToT).^11^ These approaches introduce the concept of “thought” as a sequence of tokens that represents the intermediate steps in humanlike reasoning process. By incorporating such intermediary steps, LLM reasoning moves beyond simple autoregressive token generation, enabling more sophisticated cognitive architectures like tree search^11^ and reflective reasoning.^12^

Recently, there has been a significant research trend in learning to reason,^13^ which seeks to train LLMs to master humanlike reasoning processes. A key challenge in this research direction is the lack of training data. Human annotation is often prohibitively expensive, particularly for step-by-step reasoning trajectories that have proven effective in supervising LLM reasoning.^14^ To address this issue, recent studies have shifted from human annotation to LLM-driven search algorithms. These approaches utilize external verification for reasoning problems to automatically generate accurate reasoning trajectories through trial-and-error search.^15^ More importantly, researchers have proposed to train process reward models (PRMs) on these reasoning trajectories.^16^ PRMs can provide dense, stepwise rewards that facilitate reinforcement learning for LLM reasoning. These methods combine to reduce the reliance on human annotation data, and create a “reinforced cycle” for augmenting LLM reasoning that effectively integrates “search” and “learning,” which are the two methods that can scale endlessly as predicted by Richard Sutton.^17^ Therefore, this novel paradigm enables the scaling of LLMs’ reasoning capabilities with increased train-time compute, paving the way for more advanced reasoning models.

Moreover, a recent study shows scaling up test-time compute can also improve the accuracy of LLM reasoning. Specifically, PRMs can be used to guide LLMs to evaluate and search through the intermediate “thoughts,”^18^ which encourages LLMs to generate deliberate reasoning steps during test-time computation and boosts reasoning accuracy. This approach gives rise to the test-time scaling law, which predicts that spending more tokens for deliberate reasoning at test time can improve accuracy.^13^ Therefore, the RL-driven train-time scaling and search-based test-time scaling combined to show a promising research direction to fully unleash the reasoning capabilities of LLMs, i.e., a path toward *large reasoning models*. A key milestone in this research direction is OpenAI’s o1 series,^19^ which demonstrates the effectiveness of this approach and echoes OpenAI’s vision of transitioning LLMs from *conversational AI* (level 1) to more powerful *reasoning AI* (level 2) in the five-step road map toward AGI.^20^ Several open-source projects, such as OpenR,^21^ LLaMA-Berry,^22^ and Journey Learning,^23^ are dedicated to reproducing the strong reasoning capacity of OpenAI’s o1, providing valuable insights for developing large reasoning models.

**Figure 1:**
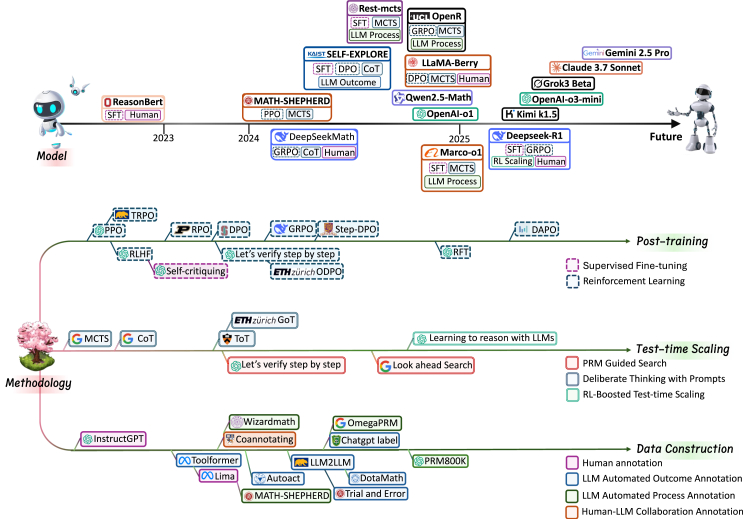
Overview of the development trajectory of large reasoning models, demonstrating how innovation in post-training, test-time scaling, and data construction techniques collectively drive progress

Figure 1 presents a conceptual taxonomy on how recent research efforts in post-training, test-time scaling, and data construction techniques collectively drive the progression toward large reasoning models. In this survey, we provide a comprehensive review of the most relevant recent literature based on the proposed taxonomy. Specifically, the section “background” offers a brief introduction to the background of LLM reasoning. The subsequent three sections delve into the key technical components driving the development of large reasoning models, i.e., “data construction: from human annotation to LLM automation” focuses on training data construction, emphasizing the shift from human annotation to LLM-driven automated search; “learning to reason: from supervised to reinforcement fine-tuning” reviews reinforcement learning methods that are pivotal for scaling LLMs’ reasoning capabilities with increased train-time compute; and “test-time scaling: from CoTs to PRM-guided search” discusses test-time scaling with a particular emphasis on PRM-guided search. After reviewing these main technical components, we perform a critical analysis on the strengths and limitations of different techniques on various task scenarios in the section “summarizing the main technical components.” Besides, we also provide an in-depth analysis on the development of OpenAI’s o1 series as well as the important open-source projects in “path toward large reasoning model,” exploring the early attempts of practically building large reasoning models. The section “other test-time enhancing techniques” summarizes additional test-time enhancement techniques, and “evaluation benchmarks” reviews reasoning benchmarks. Finally, we conclude the survey with a discussion of open problems and future research directions.

## Background

### Pretraining

As the foundational stage of training LLMs, effective pretraining is crucial for developing reasoning abilities. Before discussing pretraining for LLM reasoning, we first outline the basic process of general LLM pretraining. Through pretraining, LLMs not only acquire core linguistic knowledge but also gain diverse world knowledge, establishing a robust groundwork for the emergence of advanced capabilities and effective value alignment.^1^ Typically, LLM pretraining relies on high-quality text corpora,^24^^,^^25^ including extensive collections of web content, books, codes, and other types of data. Leveraging these rich textual corpora, LLMs are built on the transformer architecture that are trained with the next-token prediction task. After pretraining, LLMs generally demonstrate exceptional in-context learning capabilities,^26^ enabling them to generate coherent text and provide accurate answers to a wide range of questions by utilizing their vast knowledge base. Notably, the pretraining stage plays a pivotal role in cultivating the reasoning abilities of LLMs. For example, research^10^ has shown that datasets rich in code and mathematical content serve as a key foundation for developing robust reasoning skills. Following these observations, newly developed LLMs^27^ begin to introduce carefully designed synthetic data for enhancing the reasoning abilities of LLMs. During pretraining, a critical challenge lies in balancing the proportion of code and mathematical data with general text corpora to maintain strong general linguistic abilities while unlocking the reasoning potential of LLMs.

### Fine-tuning

While pretraining enables LLMs to exhibit reasoning abilities through in-context learning, fine-tuning techniques are widely employed to achieve zero-shot and improved reasoning capabilities for LLMs. Here, we first outline the basic fine-tuning process and then explore its potential for enhancing reasoning abilities. As described by Ouyang et al.,^28^ after the pretraining stage, LLMs enter a supervised fine-tuning phase (SFT), also referred to as the instruction tuning stage. The primary goal of this phase is to refine the model’s output style, ensuring its responses are aligned with human needs and real-life applications. This is achieved by training via diverse instruction datasets that reflect a wide range of everyday human interactions, typically created through extensive and carefully curated manual annotation and refinement.^29^ With the advent of ChatGPT, new methods have emerged for generating diverse instruction datasets. These include techniques that distill data directly from powerful LLMs^30^^,^^31^ and automated approaches for large-scale dataset construction from existing corpora.^32^^,^^33^ Using these well-crafted instruction-tuning datasets, the fine-tuning process continually uses the next-token prediction objective, similar to pretraining. However, unlike pretraining, fine-tuning specifically calculates the loss for the answers while generally ignoring the loss for the questions. Besides, incorporating datasets that include chain-of-thought (CoT)^10^ reasoning and mathematical problem-solving examples has been shown to significantly enhance the reasoning capabilities of LLMs,^34^ making this an area of active research. Following the practice in general aspects, most current approaches leverage data distillation from advanced large reasoning models, followed by fine-tuning to enhance the reasoning capabilities of LLMs to obtain the final large reasoning models.

### Alignment

Relying solely on direct data distillation from advanced large reasoning models limits the potential of new LLMs. A more promising approach is to use reinforcement learning for data construction and model training, which precisely corresponds to the final alignment stage in general LLM training. In the general training of LLM, the alignment phase typically involves methods such as reinforcement learning from human feedback (RLHF)^28^ to guide the model toward generating content that meets the criteria of being helpful, harmless, and honest. The goal of this phase is to enhance the safety and controllability of LLMs in the reality. Compared to the former SFT phase, this stage usually incorporates a large amount of carefully curated, manually labeled ranking data to accurately reflect human preferences.^24^^,^^25^ These data include not only correct demonstrations but also undesirable cases that should be avoided. Standard RLHF typically involves an SFT model, a reward model, and an aligned model, which are iteratively optimized using methods like proximal policy optimization (PPO).^35^ Due to the high data requirements and training costs of standard RLHF, methods like direct preference optimization (DPO)^36^ have been proposed to reduce reliance on explicit reward models. In DPO, preference loss is defined as a function of the policy to directly guide model optimization. Given the multi-step nature and complexity of reasoning problems, alignment-based post-training has become the final and most critical step in stimulating the reasoning capabilities of LLMs. By carefully decomposing the reasoning process and gradually feeding signals back to the model, various self-training methods^16^^,^^37^^,^^38^ based on reinforcement learning and preference learning have achieved notable success.

### Prompting LLMs for advanced reasoning

Humanlike reasoning is one of the most important abilities that emerge in LLMs with sufficiently large model parameters.^6^ While zero-shot reasoning may remain unreliable for some tasks, researchers have discovered various prompting techniques to enhance these capabilities. These techniques can be broadly categorized into three main approaches: step-by-step reasoning, multi-path exploration, and decomposition-based methods.

The step-by-step reasoning approach, exemplified by chain-of-thought prompting,^10^ demonstrates that explicitly showing intermediate reasoning steps significantly improves problem-solving abilities. Even simple prompts like “let’s think step by step” can effectively guide the reasoning process.^39^ This approach has been further refined through self-consistency,^30^ which generates multiple reasoning paths to arrive at more reliable conclusions, and auto-CoT,^40^ which automates the generation of effective reasoning chains.

Multi-path exploration approaches extend beyond linear reasoning by considering multiple potential solution paths simultaneously. ToT^11^ organizes alternative reasoning pathways in a tree structure, enabling systematic exploration of different solution strategies. Graph-of-thoughts^41^ further generalizes this to a graph structure, allowing for more flexible reasoning patterns and backtracking capabilities. ReAct^42^ enriches this paradigm by interleaving reasoning with action steps, enabling more dynamic interaction with external environments.

For complex problems, decomposition-based methods have proven particularly effective. Least-to-most prompting^43^ and algorithm of thoughts^44^ systematically break down complex problems into manageable components, while plan-and-solve^45^ provides strategic guidance for tackling these subproblems. These methods are especially valuable when dealing with tasks that require multiple steps or different levels of analysis.

These extensive reasoning capabilities, enhanced through structured prompting strategies, have proven particularly effective for tasks requiring careful analysis and systematic thinking, enabling LLMs to accomplish a wide variety of complex social scientifically relevant tasks. The success of these methods demonstrates that while LLMs possess inherent reasoning abilities, their full potential can be unlocked through careful guidance and structure in the prompting process.

### Agentic workflow

On top of the instruction following and in-context learning capabilities of LLMs, researchers start to design agentic workflows that program the “thinking patterns” of LLMs.^46^ Such agentic workflows allow researchers to enhance LLMs’ reasoning capability without any additional training, but it often requires more test-time compute. In-context learning^4^^,^^47^ is the ability to improve LLM’s task-specific performance by simply providing a few in-context demonstrations, enables LLMs to efficiently generalize to unseen problems without computationally expensive trainings.^26^ Although the origin of such capabilities remains a largely debatable topic, recent studies suggest in-context learning improves LLMs’ performance by allowing them to capture the label space, the distribution of input text, and the desired format of answers.^48^ Such desirable features have enabled researchers to adapt general-purpose LLMs to diverse task scenarios, such as simulating the perspective of certain demographic groups through in-context role play.^49^ Recent studies suggest effective agentic workflow can largely improve LLMs’ abilities for simulating human behavior,^50^^,^^51^ human-LLM interaction,^52^ situated instruction following,^53^ and collaborative task solving.^54^ The ability to program LLMs with agentic workflow lays the foundation of improving LLMs’ reasoning capabilities with complex cognitive architecture.

## Data construction: From human annotation to LLM automation

**Figure 2:**
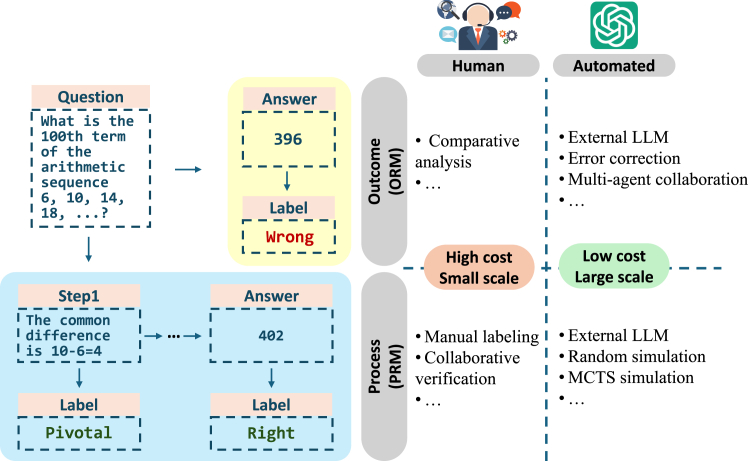
Illustrating different paradigms for annotating LLM reasoning data

Creating large-scale, high-quality reasoning datasets is crucial for enhancing the reasoning capabilities of LLMs. However, this task poses significant challenges due to its high cost. As shown in Figure 2, human annotation is widely considered of high quality but is prohibitively expensive and difficult to scale. Conversely, automating the annotation process with LLMs offers a more cost-effective alternative but faces the challenge of limited validation, particularly for step-by-step reasoning processes. In this section, we review recent research efforts in this area (summarized in Table 1), highlighting the shift from human annotation to LLM automation.

**Table 1:** Training data construction for LLM reasoning

Method	Label	Reference	Year	Task	Brief description
Human annotation	outcome	Nasution and Onan^55^	2024	text classification; semantic analysis	voting annotation
		Ouyang et al.^28^	2022	preference alignment	preference ranking
	process	Lightman et al.^14^	2023	mathematical reasoning	stepwise annotation
Human-LLM collaboration	outcome	Goel et al.^56^	2023	semantic analysis	human correction
		Wang et al.^57^	2024	text classification	human correction
		Li et al.^58^	2023	text classification; semantic analysis	task allocation; uncertainty assessment
LLM automation	outcome	Puri et al.^59^	2020	common-sense reasoning	text extraction
		Schick et al.^60^	2024	tool use	trial and error
		Kwon et al.^61^	2024	embodied tasks	synthetic augmentation
		Qiao et al.^62^	2024	common-sense reasoning; domain knowledge reasoning	multi-agent collaboration
	process	Luo et al.^63^	2023	mathematical reasoning	stronger LLM
		Wang et al.^64^	2024	mathematical reasoning	Monte Carlo simulation
		Wang et al.^65^	2024	mathematical reasoning; programming	Monte Carlo simulation
		Luo et al.^15^	2024	mathematical reasoning	MCTS simulation
LLM automation with feedback	outcome	Lee et al.^66^	2024	text classification; mathematical reasoning; domain knowledge reasoning	self-refining
		Song et al.^67^	2024	embodied tasks	contrastive learning
	process	Zhang et al.^16^	2024	mathematical reasoning; domain knowledge reasoning	MCTS simulation; self-refining

### Human annotation

The role of human annotation in constructing datasets for LLMs is indispensable. Human annotators are characterized by their meticulousness, patience, and precision, as well as their adaptability to novel scenarios and capability to handle ambiguous data effectively.^55^ Zhou et al.^29^ demonstrate that even with minimal human-annotated data, models can achieve strong performance, highlighting the critical role of carefully curated annotations in model effectiveness. Human-annotated data plays a pivotal role in enhancing the reasoning capabilities of large language models. In the context of RLHF,^28^ preference data from human annotators enables LLMs, initially trained on general text corpora, to align with intricate human values and complex ethical considerations. This generalizable approach of annotation helps in fine-tuning models for specific tasks. Building on this foundation, Lightman et al.^14^ demonstrated the efficacy of using human annotators to evaluate the reasoning quality at each step of mathematical reasoning processes, significantly improving the accuracy of LLM reasoning. This highlights how human annotation can bridge the gap between general training data and domain-specific challenges, such as complex reasoning tasks.

Enhancing reasoning capabilities in LLMs requires process supervision, where human annotators guide each step of the reasoning process.^14^ However, such supervision demands extensive human-annotated data, making it resource-intensive and unsustainable. Given that LLM training typically requires terabytes of data, the volume of which is critical for model performance, constructing datasets purely through manual annotation becomes increasingly impractical. This highlights the need for alternative approaches to improve reasoning without relying solely on human annotation. One promising approach is the collaboration between humans and LLMs for annotation, where LLMs are leveraged to accelerate the process while preserving the high quality of human-generated annotations. Specifically, the annotation process can be divided into two stages: the preannotation stage and the refinement stage. During the preannotation stage, LLMs can be employed to perform an initial round of annotations, taking advantage of a few manually provided examples for a quick and efficient setup.^56^^,^^68^ In the refinement stage, human annotators can assess the quality of LLM-generated annotations and focus on correcting only the subset of annotations with poor quality.^56^^,^^57^^,^^68^^,^^69^ To enable scalable annotation processes, recent works have increasingly focused on how to maximize automation while ensuring data quality, thus reducing human involvement without compromising the accuracy of the annotations.

### LLM automated outcome annotation

Data annotation is a challenging and resource-intensive task, particularly in scenarios requiring complex operations such as filtering, identifying, organizing, and reconstructing textual data. These tasks often are tedious and time-consuming and demand significant human effort, making them a costly bottleneck in large-scale data construction efforts.^70^^,^^71^ To address these challenges, leveraging LLMs for data annotation provides a cost-effective and efficient alternative. With context window lengths exceeding 100k tokens, LLMs can effortlessly process lengthy texts and large volumes of structured data,^72^ handling the intricate requirements of data annotation with remarkable efficiency. Their strong instruction-following capabilities^73^ enable them to flexibly accommodate diverse and complex annotation scenarios, while achieving a level of quality comparable to that of human annotators. By automating these demanding tasks, LLMs significantly reduce the reliance on human labor, streamlining the annotation process and enhancing overall productivity.^74^

LLMs are capable of handling a wide variety of automated annotation tasks, ranging from simple question-answer extraction^59^ to the inclusion of additional target information.^75^ Without human demonstrations, LLMs rely on their powerful reasoning and in-context learning abilities to independently address more complex annotation needs. For instance, Schick et al.^60^ demonstrated how LLMs can be used to construct datasets for tool usage. For each candidate position that may require an API call, the LLM is able to comprehend the logical relationships within the surrounding context, generate relevant questions, and identify the appropriate tool API to address the issue. When human demonstrations are available, LLMs can further enhance their performance by mimicking the patterns and reasoning strategies illustrated in these examples. For complex tasks, human demonstrations provide high-quality trajectories—sequences of thoughts, observations, or actions—that guide the LLMs in replicating human decision-making processes. Existing studies have shown that even zero-shot LLMs, guided by task-agnostic prompts based on human demonstrations, can perform annotation tasks effectively.^61^ Moreover, for tasks involving highly intricate and nuanced trajectories, LLMs can incorporate specialized agents, such as a plan-agent, tool-agent, and reflect-agent, to address different aspects of the annotation process, thereby further enhancing their ability to align with humanlike reasoning and behavior.^62^ These diverse capabilities extend naturally to reasoning outcome annotation tasks, where LLMs not only infer underlying logical structures but also systematically document intermediate reasoning steps and their associated conclusions. This enables the creation of annotated datasets that capture not just final outcomes but the full reasoning processes leading to them, offering richer insights for downstream applications.

Beyond annotation with human demonstrations, LLMs can independently enhance their annotation capabilities through search with feedback, a process that involves iterative refinement by learning from a dynamic environment. Failed data points can be considered a classic form of feedback, serving as valuable feedback for the model to identify weaknesses and design targeted adjustments. By self-correcting erroneous samples and generating refined training data, LLMs engage in a cycle of self-improvement that strengthens both their understanding and reasoning.^66^ Furthermore, LLMs can systematically analyze the causes of their errors, extracting key insights and encoding these as self-learned knowledge to guide future reasoning tasks.^76^ This feedback-driven approach can also involve pairing failed trajectories with successful ones based on similarity, enabling contrastive learning strategies to refine the model’s parameters. Through such iterative search and refinement mechanisms, LLMs not only address errors but also develop a more robust capacity for reasoning, enabling deeper generalization and adaptability across complex tasks.^67^

### LLM automated process annotation

In complex reasoning tasks, each step of the model’s output can significantly influence the final result, making it essential to label intermediate decisions as “correct” or “incorrect” or assign an intermediate reward, namely process annotation. However, manually labeling these steps is costly and time-consuming. For example, Lightman et al.^14^ invest massive manual efforts to produce a large-scale process annotation dataset, i.e., PRM800K, which satisfies the requirement in training an effective PRM and greatly enhances the reasoning capability of LLMs. Therefore, automated methods are increasingly needed for efficient process annotation, ensuring scalability and cost-effectiveness. Initial automated approaches hire external stronger LLMs to annotate the intermediate process generated by smaller LLMs. Furthermore, a Monte Carlo-based method reduces the reliance on external stronger LLMs and can use weaker LLMs to complete data annotation and thereby train stronger LLMs via a self-reinforced manner.

#### Annotation with stronger LLMs

As a straightforward automated labeling method, Luo et al.^63^ utilize a more powerful external model to annotate the intermediate results of a generative model’s inference process. Rather than relying on manual annotation, the method employs a pretrained, high-performance model, like GPT series, to evaluate each generated step. By leveraging the capabilities of a stronger external model, this approach enhances both the accuracy and scalability of the labeling process, making it more feasible for large-scale tasks. However, the major limitation of this approach is its reliance on the highly capable external model, which means the performance of the labeling process is ultimately constrained by the capabilities of the external model used.

#### Annotation by Monte Carlo simulation

To reduce reliance on the powerful external models, Wang et al.^64^^,^^65^ propose an improved method that avoids directly scoring the intermediate steps. Instead, their approaches use an external model to continue the reasoning for several steps from the given intermediate output and randomly repeat this simulation process multiple times. The quality of the intermediate step is then assessed based on the average outcome of these extended inferences. This Monte Carlo method has shown promising results in tasks such as mathematical problem solving and code generation.

#### Annotation by tree search simulation

The approach of using multiple-step Monte Carlo simulation with an external model to assess the quality of intermediate steps based on the average outcomes has become one of the most widely used methods for automated process annotation. To further enhance the efficiency of this method, Luo et al.^15^ propose an improvement by replacing the repeated Monte Carlo simulations with a Monte Carlo tree search (MCTS) strategy. In this improved method, multiple leaf nodes representing the final inference results are generated from the intermediate step using MCTS. The quality of the intermediate step is then evaluated based on the average outcomes of these leaf nodes. Compared to random repeated inferences, MCTS leverages tree search to improve the inference quality while also allowing leaf nodes to share high-quality parent nodes, reducing computational overhead and increasing efficiency. This method has demonstrated superior performance in mathematical problem solving, outperforming human annotations.

One step forward from the MCTS-based simulation, Zhang et al.^16^ introduces a self-refining mechanism into the process annotation. They leverage the obtained process annotations to train a PRM, which in turn improves the performance of the LLMs. The refined LLM is then used to repeat the MCTS-based simulation, generating higher-quality annotations. This iterative process, involving repeated cycles of improvement, results in progressively enhanced process annotations. This method has shown excellent performance across several tasks, including mathematical problem solving, questioning and answering, and multi-domain knowledge reasoning, demonstrating its effectiveness in continuously refining and improving the quality of the annotations through iterative enhancement.

## Learning to reason: From supervised to reinforcement fine-tuning

While pretrained models excel across various tasks, they often struggle with complex reasoning and aligning outputs with human expectations. Fine-tuning is crucial to address these limitations, refining a model’s performance on specific tasks and enhancing its reasoning capabilities. Initially, SFT is used, where models learn task-specific patterns from labeled datasets. However, as reasoning challenges grow, methods like reinforcement learning (RL) and DPO offer a more effective approach, using reward models to more efficiently align the model’s output with humanlike reasoning, fostering more coherent, responsible, and contextually aware outputs.

### Optimizing pretrained LLM: Supervised fine-tuning

Supervised fine-tuning is a learning technique that refines pretrained models’ capabilities for specific tasks or domains using labeled data, while retaining the model’s understanding of pretrained knowledge. While pretraining allows models to learn broad, general-purpose features from massive amounts of unstructured data, fine-tuning specializes the model by exposing it to smaller, task-specific datasets with clear input-output mappings.

SFT is a critical step in improving the reasoning ability of LLMs, enabling their application in downstream tasks by adapting them from general-purpose systems to domain-specific tools. For example, LLMs like GPT,^77^ BERT,^78^ and T5^79^ are pretrained on vast amounts of text data using self-supervised learning, equipping them with broad language understanding and generation capabilities. However, their outputs are not always aligned with task-specific requirements. Without fine-tuning, LLMs tend to perform poorly on certain reasoning tasks, such as object counting,^80^ satellite understanding,^81^ and engineering question answering.^82^ Through SFT, we can partially address these challenges by refining the model’s outputs based on labeled task-specific datasets.

However, the direct application of SFT may not fully explore the model’s reasoning capabilities in the desired domains, particularly in tasks that require more complex decision-making or multi-step problem-solving. The introduction of CoT techniques^10^ has revolutionized the SFT process, by explicitly training the model to generate intermediate reasoning steps before arriving at an answer. With CoT-based SFT, LLMs are encouraged to generate intermediate reasoning steps explicitly, thus enhancing their reasoning ability to tackle tasks that require more structured and organized thoughts. For instance, ReasonBERT^83^ shows that fine-tuning models with reasoning chains significantly enhances their performance on tasks such as math word problems and logical reasoning by incorporating step-by-step reasoning processes. Another key study^84^ investigates how fine-tuning models with reasoning improves their interpretability and reduces errors in complex decision-making scenarios by generating more transparent, stepwise thought processes. By fine-tuning with CoT, models not only improve their final answers but also enhance their ability to “think through” the problem, providing clearer insights into the model’s reasoning process.

Despite the diverse methods and outstanding performance of SFT, it comes with several limitations. First, SFT heavily relies on high-quality labeled datasets, which can be expensive and time-consuming to curate, especially for niche domains or tasks requiring expert annotations. Second, SFT may lead to catastrophic forgetting, where the model loses some of its pretrained general-purpose knowledge during the fine-tuning process, reducing its utility for tasks reasoning outside the fine-tuning domain. Finally, the computational cost of fine-tuning large-scale models can still be prohibitive, even with parameter-efficient approaches, posing challenges for organizations with limited resources. Addressing these limitations requires careful dataset curation, regularization techniques, and the exploration of alternative methods, such as prompt tuning or multi-task fine-tuning, to balance task specialization and generalization.

### Optimizing pretrained LLM: Reinforcement learning

**Figure 3:**
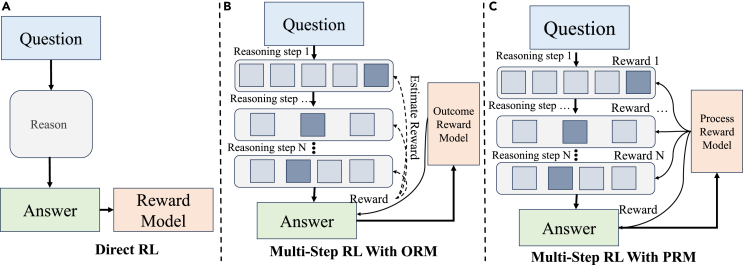
Reward models for train-time reinforcement learning that boosts LLM’s reasoning capability

Due to the high reliance on expensive, high-quality labeled datasets, and high computational costs of SFT, reinforcement learning has emerged as a powerful alternative framework for training models to master reasoning processes. Unlike supervised learning, RL enables models to learn through trial and error reward signals, discovering optimal strategies for achieving specific objectives. As shown in Figure 3A, the model takes action based on its current state and receives feedback in the form of a reward signal. This feedback guides the model to update its parameters over time, optimizing for cumulative rewards.

#### Classic reinforcement learning

RL has become a critical step in the development of LLMs. In RL framework, the parameters of LLMs are updated based on the rewards for their actions. Specifically, the value function or Q-function is updated based on the feedback of reward model, attributing the credit for an action’s outcome entirely to its immediate effect. This approach simplifies the framework, making it conceptually straightforward while enhancing the model’s ability to respond effectively. Two key methods currently dominate RL training for LLMs: RLHF and reinforcement learning from AI feedback (RLAIF).

Ouyang et al.^28^ use RLHF to align LLMs with human intent. Besides, by fine-tuning GPT-3 on human-labeled demonstrations and rank comparisons, they develop a reward model predicting human annotator’s preferences. It effectively aligns trained LLMs with human preferences, outperforming GPT-3 in reasoning and instruction-following despite being smaller. Bai et al.^85^ also leverage RLHF to create helpful and harmless language models. Following a helpful, honest, and harmless framework, they fine-tune a base model, train a preference model with rejection sampling, and iteratively refine it with human feedback. This process produces AI assistants that excel in NLP tasks and demonstrate strong ethical reasoning.

To reduce the reliance on large human-labeled datasets, Bai et al.^86^ propose constitutional AI, a framework for training AI assistants to be helpful and harmless using principles instead of expensive human feedback. The process includes two phases: supervised learning and RLAIF. In the supervised phase, the model critiques and refines its outputs based on constitutional principles, creating a fine-tuning dataset. In the RLAIF phase, the model generates self-assessments to guide training, bypassing the need for human-labeled data on harmfulness. Ramamurthy et al.^87^ focus on using RL to align LLMs with human preferences. They introduce RL4LMs, a library for RL-based fine-tuning, and the GRUE benchmark, which evaluates models using reward functions reflecting human preferences. To address training challenges, they propose the natural language policy optimization algorithm, which stabilizes training by constraining token sampling. This work provides a strong foundation for integrating RL into LLM fine-tuning for improved alignment and performance.

#### Direct preference optimization

Classic RL methods rely on training a reward model to score outputs based on human preferences. While DPO streamlines this process by directly leveraging preference data without requiring an explicit reward model. Instead of optimizing a complex reward function, DPO uses pairwise preference comparisons, i.e., data indicating which of two outputs is preferred by humans. This direct approach simplifies the learning pipeline while preserving the alignment benefits of RL-based methods, which is often simpler and more effective. Rafailov et al.^36^ introduce DPO, a novel framework for aligning language models, which directly optimizes the policy to align with human preferences through a simple classification loss. By parameterizing the reward model to derive an optimal policy in closed form, DPO eliminates the need for sampling and extensive hyperparameter tuning during fine-tuning. Experiments show that DPO matches or surpasses RLHF methods like PPO in tasks such as sentiment control, summarization, and dialogue generation, while being more stable, computationally efficient, and effective in producing reasoning outputs. Amini et al.^88^ propose direct preference optimization with an offset (ODPO), an extension of DPO for aligning language models with human preferences. ODPO improves upon DPO by considering the degree of preference between responses rather than treating all preference pairs equally. It introduces an offset in the likelihood difference between preferred and dispreferred responses, proportional to their quality difference. This approach not only improves alignment but also strengthens the model’s reasoning capability, particularly in tasks such as sentiment control, toxicity reduction, and summarization. Experiments demonstrate that ODPO achieves better alignment and responsible behavior, especially when preference data are limited.

In conclusion, the RL and DPO methods offer a straightforward and effective method for fostering reasoning ability in LLMs. By focusing on immediate rewards following each action, these methods also align models with human preferences. The emphasis on short-term feedback simplifies the learning process, avoiding the complexities of credit assignment across long sequences. This streamlined approach is particularly well-suited for real-time applications and tasks requiring clear, concise reasoning, ultimately strengthening the ability of LLMs to deliver coherent and ethical outcomes.

### Enhancing multi-step reasoning with outcome reward model

For complex reasoning tasks, such as mathematical problem-solving, LLMs need to perform multi-step reasoning like chain-of-thought to ultimately reach an accurate solution. In these tasks, the reward feedback is typically only available after all the reasoning steps are completed and the final solution is obtained. As shown in Figure 3B, this is known as the outcome reward model (ORM). In such cases, the key to improving the LLMs’ reasoning capability lies in distinguishing the correctness and importance of intermediate reasoning steps based on the outcome rewards.

#### Classic reinforcement learning

ReFT^89^ applies the PPO^35^ method from RLHF^28^ to reasoning tasks. Based on the outcome reward model, the value function in PPO is able to infer the contribution of intermediate reasoning steps. Compared to supervised fine-tuning, ReFT is capable of learning more diverse reasoning paths, exhibiting stronger generalization abilities in reasoning tasks. However, VinePPO^90^ discovers that the value network trained with ORM in PPO exhibits significant bias when identifying the value of intermediate reasoning steps, a well-known challenge in RL called the credit assignment problem. To address this issue, VinePPO abandons the value network in PPO and instead employs a Monte Carlo sampling method to compute unbiased estimates of the value function. Experimental results demonstrate that VinePPO consistently outperforms typical PPO in mathematical reasoning tasks. Critical plan step learning (CPL) is a method designed to enhance LLM’s generalization in reasoning tasks by searching within high-level abstract plans.^91^ CPL employs MCTS to explore different planning steps in multi-step reasoning tasks and utilizes Step-APO to learn critical plan steps. This approach enables models to learn more diverse reasoning paths, thereby improving generalization across various tasks. Subsequently, the model iteratively trains the policy and value models to further enhance performance. During each iteration, the policy model generates plan steps and final solutions, while the value model evaluates the quality of intermediate steps. Training data, generated by MCTS, is used to update both the policy and value models.

#### Direct preference optimization

In the task of mathematical reasoning, directly employing the DPO^36^ method for preference optimization yields suboptimal results due to the presence of lengthy reasoning steps in the preference data. Amini et al.^88^ introduced ODPO, which refines DPO by taking into account the degree of preference between responses instead of treating all preference pairs as equal. ODPO has achieved significant improvements over DPO in mathematical reasoning tasks.

In summary, the primary challenge of training based on outcome rewards lies in distinguishing the correctness and importance of intermediate reasoning steps. Current methods, primarily based on Monte Carlo sampling or Monte Carlo tree search, offer advantages in estimating the significance of these intermediate steps, though the computational cost during search remains high. Existing work has primarily focused on mathematical or other reasoning problems, where the final solutions can be easily verified. These methods can be extended to a wider range of reasoning tasks, including those where the solutions are difficult to validate. A potential approach is to learn a reward model based on human annotation data and use it to judge the quality of the final solution. Based on the final score provided by the reward model, Monte Carlo sampling or search techniques can then be employed to further improve performance.

### Enhancing multi-step reasoning with process reward model

PRM-driven reinforcement learning represents a significant advance in LLM reasoning, emphasizing the evaluation of intermediate steps rather than solely focusing on end-state outcomes. As shown in Figure 3C, the reward of PRM is distributed across each reasoning step, rather than being concentrated at the final outcomes. By providing nuanced feedback throughout the reasoning trajectory, PRM enables models to optimize behavior with greater alignment to human preferences and complex task requirements. This approach is crucial for tasks that involve sequential decision-making, where intermediate steps or decisions are of significance for the final goal. We explore PRMs’ evolution and highlight their role in improving reasoning by providing step-level rewards during complex tasks.

#### Classic reinforcement learning

A series of recent works apply PRMs for mathematical or logical reasoning, since a seminal work from OpenAI^14^ has proven the importance of process reward. SELF-EXPLORE^92^ uses PRMs to enhance mathematical reasoning by identifying and addressing “first pits,” which are the initial incorrect steps in problem-solving. By rewarding steps that correct such errors, PRMs enable self-supervised fine-tuning without requiring extensive human annotations. This model achieves significant improvements in accuracy on mathematical benchmarks like GSM8K and MATH by leveraging step-level fine-grained feedback. MATH-SHEPHERD^93^ introduces a PRM framework designed for step-by-step verification and reinforcement in mathematical reasoning tasks. By automating process supervision through MCTS-inspired methods, MATH-SHEPHERD eliminates the need for human annotations while ensuring high accuracy in multi-step problem-solving. PRMs are employed to reinforce logical progression and correctness, resulting in improved performance on benchmarks like GSM8K and MATH. DeepSeekMath integrates PRMs via group relative policy optimization (GRPO),^94^ an RL algorithm that optimizes step-level rewards. PRMs are used to enhance mathematical reasoning and reasoning consistency across domains. By focusing on intermediate reasoning steps, DeepSeekMath achieves state-of-the-art performance on several benchmarks, showcasing the power of PRMs in mathematical domains. Scaling automated process verifiers introduces Process Advantage Verifiers (PAVs), a PRM variant, to evaluate step-level progress in problem-solving.^95^ PAVs use step-level supervision to improve the efficiency and accuracy of search algorithms and reinforcement learning. By focusing on steps that make meaningful progress toward a correct solution, PAVs enable substantial gains in sample efficiency, compute efficiency, and reasoning accuracy compared to outcome reward models. This demonstrates the importance of fine-grained process rewards in scaling LLMs’ reasoning capabilities.

#### Interactive process reward models

PRMs are also applied to interactive tasks, such as conversation and multi-turn question answering. ArCHer employs a hierarchical RL approach using PRMs to train agents for multi-turn, long-horizon tasks.^96^ It implements a dual-layer system: a high-level value function evaluates utterance-level rewards, while a low-level PRM optimizes token-by-token generation within each turn. This hierarchical structure ensures more effective credit assignment and allows for nuanced training of language models to handle multi-turn interactions and reasoning tasks. The use of PRMs enables ArCHer to scale efficiently, achieving significant gains in sample efficiency and performance across agent tasks. Multi-turn reinforcement learning from preference human feedback^97^ integrates PRMs into multi-turn reinforcement learning to optimize long-term objectives with human feedback. The multi-turn preference optimization (MTPO) algorithm compares entire multi-turn interactions to generate preference signals, where PRMs are used to assign step-by-step rewards. This enables LLM agents to align behavior with long-term goals, improving overall performance in dynamic, multi-turn tasks such as conversations and strategic decision-making.

#### Direct preference optimization

Several recent studies leverage MCTS to enable the optimization of multi-step reasoning tasks through direct preference optimization.^16^^,^^98^^,^^99^^,^^100^ For instance, SVPO^98^ employs MCTS to automatically annotate step-level preferences for multi-step reasoning tasks. From the perspective of learning to rank, it trains an explicit value model to replicate the behavior of an implicit reward model. Furthermore, SVPO integrates the explicit value model with DPO, where the value model not only aids the policy model in navigating more efficient reasoning paths but also guides preference learning. However, these works primarily focus on first collecting preference data or training a reward model and then performing policy optimization based on static data and the pretrained reward model. Xie et al.^98^ advanced these approaches by integrating data collection and policy preference optimization into an iterative process. This method can be considered as an online version of direct preference optimization, where the updated policy is iteratively utilized to collect preferences through MCTS.

The evolution of multi-step RL techniques for LLMs reflects a transition from sparse outcome-based feedback to detailed process-oriented supervision. PRMs now stand as the centerpiece of the progression of LLMs’ reasoning capability, offering nuanced, step-level rewards that drive substantial improvements in reasoning tasks. Future research may focus on refining these models and expanding their applicability across diverse task domains.

### Reinforcement fine-tuning

Reinforcement fine-tuning (RFT)^101^ is a technique recently proposed by OpenAI for customizing expert LLMs tailored to specific vertical domains. Currently, RFT remains part of a research program and the technical details have not been fully released. Available information suggests RFT utilizes a small amount of preference data provided by users along with a grader model to evaluate LLM’s output. This technique enables iterative optimization of the LLM’s multi-steps reasoning capabilities. As a result, RFT technique can enhance LLM’s strategy of reasoning through similar problems in the optimized domains.

#### The grader model

RFT introduces the concept of a grader model to assess the outputs of LLMs. Considering that reinforcement learning training typically requires a reward model to provide feedback, the grader is likely analogous to a reward model, transforming textual inputs (e.g., questions and answers) into scalar values of reasoning quality. This suggests that the grader could act as a reward model trained on user-provided preference data, potentially operating as either an outcome reward model or a process reward model.^14^

#### Data efficiency

In OpenAI’s live sessions, it was mentioned that RFT can enable learning in new domains with as few as dozens of user preference data. This suggests that RFT facilitates the exploration of diverse reasoning paths to address tasks based on limited preference data. Such an approach demonstrates remarkably high sample efficiency while mitigating the risk of overfitting.^102^

#### Training stability

The stability of reinforcement learning training is a notoriously difficult problem that presents significant challenges to its broader application. Variations in random seeds or adjustments to certain hyperparameters can greatly impact the training outcomes of RL. In the context of the RFT project, OpenAI has announced plans to make this technology available to the public via APIs, enabling users to fine-tune domain-specific expert models using their own data. This claim potentially indicates that RFT has achieved a level of stability sufficient for reliably fine-tuning language models using RL techniques.

## Test-time scaling: From CoTs to PRM-guided search

### Elicit deliberate thinking with prompts

Beyond train-time optimization through techniques such as reinforcement learning, researchers have discovered that test-time prompting techniques like chain-of-thought and ToT can further enhance LLMs’ capabilities.^10^^,^^30^ While simply asking models for direct answers often yields suboptimal results, guiding them through explicit reasoning processes at test time significantly improves their performance.^39^ These prompting strategies have shown remarkable effectiveness across various domains, from mathematical reasoning to complex decision-making tasks.^42^^,^^43^ The emergence of structured prompting methods like ReAct and least-to-most prompting has demonstrated that LLMs can benefit from explicit guidance in organizing their thought processes, leading to more reliable and interpretable outputs.^40^ Although these approaches typically increase token consumption and computational overhead, they provide a compelling complement to train-time methods by enhancing LLMs’ reasoning capabilities and solution accuracy without requiring model parameter modifications.^11^^,^^41^ This suggests a promising direction for improving LLM performance through sophisticated test-time interventions rather than solely relying on model architecture or training modifications.

### PRM-guided search

**Figure 4:**
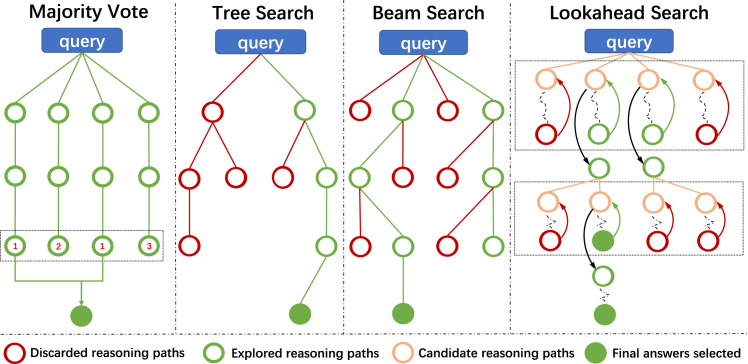
Diagrams of different search algorithms for test-time reasoning enhancement

As previously mentioned, PRM marks a significant shift from sparse outcome-based feedback to detailed process-oriented supervision. Moreover importantly, PRM can also be utilized during the test-time phase, where it can further boosts the model’s reasoning capabilities. OpenAI’s o1 models stand as a prominent example of the advanced application of PRM. The new test-time scaling laws suggest that inference capabilities can be effectively enhanced by increasing test-time compute, providing a clear direction for the future development of LLMs. We introduce some methods applied during the inference phase, as shown in Figure 4. Red hollow circles represent the reasoning paths discarded during the algorithm’s exploration process in the inference phase, green hollow circles signify the reasoning paths adopted during exploration, and green solid circles mark the endpoints of the reasoning paths once the correct answer is identified.

#### Majority vote

Majority vote^30^ is one of the most straightforward strategies to generate one final answer from dense test-time compute. During inference, each inference trace produces a prediction for a given input. The basic idea is to select the answer that most inference traces accord with. The predictions from all the models are then aggregated, and the class that appears the most (the “majority vote”) is selected as the final output: $f*=argmax_{f}\sum_{y}I_{final\_ans\left(y\right)=f}$, where I is the indicator function and y is each evaluation trace.

#### Tree search

Tree search^103^ is a classic algorithm that systematically explores different choices by recursively constructing a search tree. It is commonly used in complex decision-making problems, such as board games and planning tasks. Monte Carlo tree search is one of the most widely used tree search methods. It consists of four main steps: selection, expansion, simulation, and backpropagation. By progressively expanding the search space, MCTS incrementally improves decision-making. Tree search has already been applied in some LLM inference tasks, achieving notable success. For instance, the ToT framework^11^ enables LLMs to consider multiple reasoning paths structured as a tree. It incorporates self-evaluation to make thoughtful decisions, determining the optimal course of action for the next step. This approach significantly enhances the performance of model inference.

#### Beam search

Beam search^104^ is a heuristic search algorithm that improves upon greedy search and is widely used for generating high-quality output sequences in LLMs. Its core mechanism involves retaining a fixed number of the most promising candidates, known as the “beams” (with a beam size of k), at each step of the generation process. The selection criterion is local and myopic: at any step t, paths are scored based on their cumulative probability up to that point. By maintaining top k candidates instead of one, beam search explores a broader search space than greedy search, reducing the risk of premature commitment to a suboptimal path. Beam search is widely applied in LLM inference. BART^105^ is a successful application of beam search, demonstrating its outstanding effectiveness in text generation tasks.

#### Look-ahead search

In contrast to the myopic nature of beam search, look-ahead search^18^ employs a non-local, anticipatory scoring mechanism to enhance LLM inference. Instead of relying on the past cumulative score, it evaluates each candidate path at step t based on its estimated future potential. This is achieved by performing a forward simulation, or “rollout,” for a predetermined number of future steps (n steps). During this simulation, a pretrained and frozen PRM provides a score for each action taken. The cumulative score from this *n*-step simulation serves as a look-ahead score, which informs the pruning decision at the current step t. By grounding decisions in the outcomes of simulated futures, this strategy can circumvent the short-sighted pitfalls of standard beam search. This deeper evaluation, however, comes at the cost of significantly higher computational demand, which may impact performance when resources are constrained.

## Summarizing the main technical components

### Comparative analysis of different techniques

In the previous sections, we have introduced the key technical components that drive the progression toward large reasoning models, including dataset construction, post-training, and test-time scaling techniques. Recent research efforts have proposed multiple technical solutions for each key component. Here, we perform a critical analysis to compare strengths and limitations of each solution option and discuss their suitable application scenarios. In terms of dataset construction (Table 2), the integration of human annotation with LLM automation strikes a balance between annotation quality and efficiency, where human involvement ensures high-quality data while LLM automation significantly enhances speed and scalability. In the post-training phase (Table 3), supervised fine-tuning and reinforcement learning balance training stability with the generalization ability of LLM reasoning, as supervised fine-tuning provides controlled, stable improvements, while reinforcement learning enhances the model’s capacity for handling diverse and complex reasoning tasks. As for test-time scaling (Table 4), the use of varying prompting techniques and search algorithms offers a trade-off between computational costs and the LLM’s ability to perform accurate reasoning, where more complex strategies improve reasoning depth but come with higher computational costs. Overall, the choice of techniques reflects deliberate trade-offs, each tailored to the specific demands of different application scenarios. Future developments may further optimize these trade-offs or aim for breakthroughs that achieve simultaneous improvements across multiple dimensions, thereby driving the continued advancement of LLMs’ reasoning capabilities.

**Table 2:** Comparative analysis of different methods for dataset construction

Method	Reference	Strengths	Limitations	Typical tasks
Human	Lightman et al.^14^; Ouyang et al.^28^; Nasution and Onan^55^	high-quality annotations	time-consuming; human subjective bias	preference alignment
Human-LLM collaboration	Goel et al.^56^; Wang et al.^57^; Li et al.^58^	enhanced efficiency	model bias	textual analyses; semantic analysis
LLM automation	Luo et al.^15^; Puri et al.^59^; Luo et al.^63^; Wang et al.^64^	high efficiency; great scalability	model bias; lacking human oversight	math reasoning; programming
LLM automation with feedback	Zhang et al.^16^; Lee et al.^66^; Song et al.^67^	self-refined annotations	model bias; complex mechanism	math reasoning; domain-specific reasoning

**Table 3:** Comparative analysis of different methods for post-training

	Method	Reference	Strengths	Limitations	Typical tasks
Training algorithm	supervised fine-tuning	Zhang and Wang^80^; Mall et al.^81^; Wang et al.^82^	controllable training process	reliance on labeled data; limited generalizability	math reasoning; domain-specific reasoning
	direct RL	Ouyang et al.^28^; Bai et al.^85^; Ramamurthy et al.^87^	no reliance on labeled data; simple reward model	limited reasoning ability	preference alignment
	multi-step RL	Kazemnejad et al.^90^; Hwang et al.^92^; Shao et al.^94^	no reliance on labeled data; high reasoning capability; generalize to unseen tasks	unstable training process; high computational cost; complex reward model	math reasoning; domain-specific reasoning
	direct policy optimization	Rafailov et al.^36^; Amini et al.^88^; Chen et al.^99^	reduced computational cost; simple reward model	reliance on labeled data	preference alignment; math reasoning
Reward model	outcome reward (ORM)	Ouyang et al.^28^; Amini et al.^88^; Kazemnejad et al.^90^	low reliance on labeled data	sparse process reward	textual analyses; math reasoning
	rule-based reward	Guo et al.^106^	low reliance on labeled data; interpretable design	sparse process reward	math reasoning
	process reward (PRM)	Wang et al.^93^; Shao et al.^94^; Setlur et al.^95^	dense reward signal	reliance on labeled data; complex model design	math reasoning

**Table 4:** Comparative analysis of different methods for test-time scaling

Method		Reference	Strengths	Limitations	Typical tasks
Deliberate prompts	CoT	Wei et al.^10^; Zhang et al.^40^	complex problem-solving; explicit reasoning process	single reasoning path	math reasoning; domain-specific reasoning
	ToT	Yao et al.^11^	multiple paths comparison; backtracking and refinement	high computational cost	math reasoning; domain-specific reasoning
PRM-guided search	majority vote	Wang et al.^30^	multiple paths comparison; simple design	high computational cost	math reasoning; common-sense reasoning
	MCTS	Wang et al.^64^^,^^65^	multiple paths comparison; random path exploration	complex reward model; complex algorithm design	math reasoning; domain-specific reasoning
	beam search	Lewis et al.^105^	multiple paths comparison; improved greedy search	complex reward model; partial solution discard	textual analyses
	look-ahead search	Snell et al.^18^	multiple paths comparison; compare by simulation	high computational cost; complex reward model	math reasoning

### Case study of recent development toward large reasoning models

**Figure 5:**
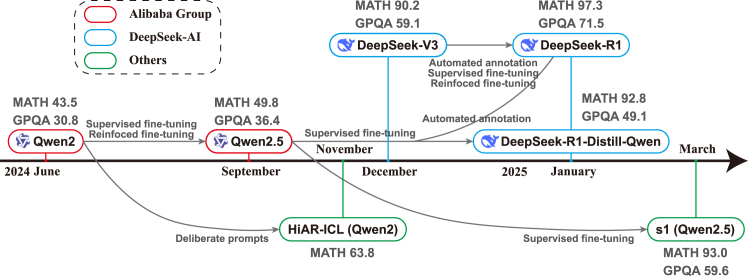
Evolution trajectory of Qwen series and DeepSeek series LLMs

To better clarify the aforementioned methods and provide a context to understand them, Figure 5 illustrates the evolutionary trajectories of the Qwen series,^25^^,^^107^ the DeepSeek series,^106^^,^^108^ and several derivative works based on these models.^109^^,^^110^ Notably, the progression from Qwen2^107^ to Qwen2.5^25^ and from DeepSeek-V3^108^ to DeepSeek-R1^106^ underscores the critical role of post-training techniques, particularly supervised fine-tuning and reinforcement learning, which substantially boost the reasoning capabilities of LLMs. Moreover, LLMs with enhanced reasoning skills can be leveraged for automated dataset construction, enabling the generation of large-scale, high-quality reasoning trajectories for training other models. For instance, the Qwen2.5 models have been further improved through fine-tuning on data labeled by DeepSeek-R1^106^ or other high-performing reasoning LLMs, leading to the development of the DeepSeek-R1-Distill-Qwen series and the s1 models.^109^ In addition, the use of deliberate prompts strengthens the reasoning capabilities of the Qwen2 series, particularly in mathematical tasks.^110^ Taken together, these examples illustrate a road map for advancing LLMs’ reasoning capabilities through a synergistic combination of dataset construction, post-training, and test-time scaling techniques.

## Path toward large reasoning model

### Development of OpenAI’s o1 series

In September 2024, OpenAI released o1, a groundbreaking language model that represents a significant advance in AI reasoning capabilities, particularly excelling in complex tasks like mathematics, coding, and scientific problem-solving. On December 20, 2024, OpenAI opened testing applications for o3, an upgraded version of o1,^111^ which is considered to have doctorate-level intelligence.^112^ These models achieve remarkable results across various challenging benchmarks, including scoring at the gold medal level in International Mathematics Olympiad^113^ and matching PhD-level performance in physics, chemistry, and biology questions.^114^ Extensive evaluations show distinct reasoning patterns of o1 series through systematic analysis of its basic reasoning capabilities. We list the key findings of existing research as follows.

#### Effective knowledge integration

Initial comprehensive evaluations^19^ demonstrate o1’s structured analytical approach and knowledge integration in fundamental problem solving tasks, achieving 83.3% success rate in competitive programming through step-by-step logical deduction, where the model demonstrates clear ability to use its knowledge to decompose complex problems and follow formal derivation processes. The model’s structured understanding and interconnected knowledge application is further evidenced in specialized fields like radiology and chip design, where accurate diagnosis and complex circuit analysis require integration of multiple domain concepts. Systematic assessments^115^ quantitatively validate this pattern, showing 150% of human-level performance in structured analytical thinking and computational reasoning tasks. This advantage is particularly prominent in scenarios requiring knowledge integration across domains, such as applying physical principles to biological systems or combining statistical methods with domain-specific constraints, indicating a fundamental capability in knowledge synthesis and application.

#### Systematic problem decomposition

o1 maintains consistent performance across tasks of varying complexity levels, showing systematic problem decomposition in handling increased difficulty. In mathematical reasoning, detailed studies^116^ show its systematic problem decomposition approach, achieving near-perfect scores on the Dutch Mathematics B exam through structured solution steps. The model demonstrates ability to identify key mathematical principles, construct formal proofs, and verify solution validity step by step. This consistency extends to more complex scenarios, as validated by research^117^ on 105 science and math problems of increasing difficulty, where the model maintains high accuracy even as problem complexity increases in terms of both conceptual depth and computational requirements. In programming tasks, this pattern is further demonstrated through systematic debugging^118^ on the QuixBugs benchmark, where o1 maintains consistent performance across bugs of varying complexity through a structured three-step approach: error identification, root cause analysis, and targeted correction.

#### Reliable and coherent reasoning in complex tasks

The model’s reasoning adapts effectively across different problem types, always showing consistency of reasoning chains in various tasks. In planning tasks, PlanBench evaluations^119^ demonstrate its systematic handling of both deterministic and probabilistic scenarios, showing significant improvement in constraint satisfaction and state management. The model shows particular strength in handling problems with incomplete information and dynamic constraints, maintaining consistent performance in both standard and rare task variants.^120^ This adaptability indicates robust generalization capabilities across different problem formulations. Studies on complex planning^121^ further show o1’s ability to maintain reasoning coherence in long-horizon tasks, effectively managing extended dependency chains and context transitions. This is evidenced by its performance in multi-step planning problems where intermediate goals must be correctly sequenced and dependencies carefully managed, demonstrating advanced capabilities in temporal reasoning and causal understanding.

#### New scaling laws for large reasoning models

Empirical studies demonstrate o1’s distinctive scaling patterns in both training and inference phases. During training, the model’s large-scale reinforcement learning algorithm teaches it to think productively using chain of thought in a highly data-efficient process.^13^ Research^18^ shows that through optimized test-time computation strategies, the model achieves significant performance improvements across various reasoning tasks. Comprehensive evaluations^19^^,^^115^ reveal that o1’s reasoning capabilities can be effectively enhanced through advanced computation allocation during inference, particularly in complex problem-solving scenarios. The constraints on scaling this approach differ substantially from those of LLM pretraining, with performance consistently improving with more time spent thinking.^13^ This is evidenced in programming tasks, where allowing 10,000 submissions per problem enables the model to achieve significantly better results, scoring above the gold medal threshold even without test-time selection strategies. The model’s ability to effectively utilize additional computation resources during both training and inference suggests a fundamental advance in reasoning architecture, demonstrating particular strength in scenarios where traditional approaches might require significantly larger model sizes.

### Open-source attempts at large reasoning models

Open-source frameworks have also made substantial strides in developing advanced reasoning capabilities for LLMs. These frameworks serve as invaluable references for researchers and developers aiming to replicate or approximate the reasoning strengths of proprietary models like OpenAI’s o1. In this section, we introduce four significant open-source efforts, each of which employs distinct strategies to enhance LLM reasoning (summarized in Table 5). By exploring their unique implementations, we aim to provide insights into the diverse methodologies used to reinforce reasoning abilities in LLMs.

#### The OpenR project

The project claimed that it is the first open-source framework to explore the core methods of OpenAI’s o1 model with reinforcement learning techniques. The core of OpenR^21^ replication is to construct step-by-step reasoning data, where the more precise and fine-grained feedback is obtained instead of purely final answers. The automated data augmentation algorithm OmegaPRM^15^ is adopted by selecting reasoning trajectories from a constructed search tree. Based on the augmented process data with supervision on each reasoning step, a process reward model is further trained in a supervised learning scheme, based on a pretrained Qwen2.5-Math-7B-Instruct model.^25^ The PRM can be directly deployed during test-time compute, integrated with either majority-vote, best-of-N, or beam-search methods. It can also be utilized to finetune LLM within the post-training stage using RL. Experiments are conducted to demonstrate the effectiveness of the PRM in test-time compute and post-training each.

#### Rest-MCTS∗

Rather than training PRM and the fine-tuned policy model separately, the authors of Rest-MCTS∗ instead integrate these two updates within one mutual self-training loop.^16^ Process reward as supervision for PRM training and reasoning traces for policy model training are collected in advance, based on a similarly designed MCTS algorithm. Then the iterative training process starts based on initial policy π and initial PRM values $V_{\theta }$. The policy further iteratively performs the MCTS and generates solutions, while the values influence the tree search process. The updates to the PRM and policy model complement each other iteratively.

#### LLaMA-Berry

The LLaMA-Berry^22^ project directs its focus on optimizing reasoning abilities at the inference stage, leveraging the LLaMA-3.1-8B architecture to deliver more sophisticated real-time reasoning adjustments. It employs a unique pairwise optimization approach that combines Monte Carlo tree search with self-refine (SR-MCTS), allowing the model to dynamically explore and refine solution paths during inference. This configuration grants LLaMA-Berry a high level of adaptability, enabling it to tackle complex, open-ended reasoning tasks efficiently and flexibly. A key component of this framework is the pairwise preference reward model (PPRM), which evaluates solution paths in pairs, ensuring that high-quality reasoning paths are prioritized. LLaMA-Berry’s enhanced borda count (EBC) then consolidates these preference rankings to guide the model’s decision-making, further enhancing its inference-stage sophistication. This robust architecture positions LLaMA-Berry as a leading example of inference-focused reinforcement, distinguishing it from O1 Replication Journey’s training-centric approach.

#### rStar-Math

The rStar-Math^122^ project aims at developing strong math reasoning ability based on small LLMs with self-evolution. Generally, it adopts similar idea on utilizing MCTS to collect high-quality process reasoning data. Differently, rStar-Math designs a novel PPM, which identifies the reasoning choices that are more favored, and thus serves as a proxy of the process supervision. Furthermore, it includes four iterations to obtain better models. In each iteration, a new smaller language model and a new PPM is fine-tuned, based on the reasoning data collected from the last round.

#### DeepSeek-R1

DeepSeek recently released a powerful open-source reasoning model with 671B parameters, achieving performance comparable to OpenAI’s o1 model. The official report discusses several limitations of using PRM, including challenges in defining fine-grained general reasoning steps, inaccuracies in labeling intermediate steps, and risks associated with reward hacking. In response to these challenges, DeepSeek-R1^106^ employs rule-based rewards for training the policy model rather than relying on PRM. Furthermore, the report highlights that MCTS is currently less desirable due to the exponentially large search space compared to traditional games like chess or Go, and the inconsistent quality of value models. Instead, DeepSeek-R1 uses LLMs to generate reasoning data in a chain-of-thought manner, followed by human supervision and rule-based improvements to enhance data quality. The complete model parameters are open-sourced for free download and have been utilized to distill smaller LLMs.

In-depth comparative analysis between these open-source frameworks is shown in Table 5. We have the following observations:

(1) In terms of specific model designs, smaller-scale open-source frameworks, such as OpenR, Rest-MCTS∗, and LLaMA-Berry (each with around 7B parameters), have focused on designing automatic reasoning data generation methods and training gradient-based reward models. These approaches enable better supervision of intermediate reasoning processes for the reasoning models. In contrast, DeepSeek-R1, as an industrial-scale reasoning model, employs a semi-automatic data construction approach, i.e., augmenting data generated by existing LLMs with human annotations. It also adopts a rule-based reward model that does not require additional training.
(2) In terms of the target area, most smaller-scale frameworks focus on math reasoning or math/science Q&A testbeds, where reasoning steps have smaller diversity and are more straightforward to interpret. The associated code and data are open sourced. In contrast, DeepSeek-R1 targets tasks from broader domains requiring general-purpose reasoning capabilities.
(3) In terms of the accessibility and usability, all smaller-scale frameworks have released the core code bases, where some also revealed the data and model weights. Besides, the models with parameter scales smaller than 8B are relatively easier to deploy on commercial platforms. In contrast, although DeepSeek-R1 has made its full 671B parameters publicly available, its code and data used for training are not shared.

**Table 5:** Comparative analysis of currently available open-source large reasoning models

	OpenR	Rest-MCTS∗	LLaMA-Berry	rStar-Math	DeepSeek-R1
Reasoning data generation	auto-generation via MCTS	auto-generation via MCTS	auto-generation via MCTS	auto-generation via MCTS	LLM generation with human supervision
Reward model (RM)	PRM	PRM	pairwise preference RM	process preference model	rule-based RM
RM training	SFT	SFT	DPO	SFT	no training
Policy training	GRPO	SFT	SFT	SFT	GRPO
Search strategy	best of N/beam search/MCTS	MCTS	MCTS	MCTS	not mentioned
Iteration	1 round	2 rounds	1 round	4 rounds	2 rounds
Accessibility	code/data	code/data/weights	code/data	code	weights
Target area	math	math/science and engineering	math/science & engineering	math	general domains
Parameter scale	7B for policy/1.5B for RM	6–8B for both	8B for policy/2B for RM	7B for policy/7B for RM	671B
Base model	Qwen2.5-1.5B-Math-Instruct/Qwen2.5-Math-7B-Instruct	LLaMA3-8B-Instruct	LLaMA-3.1-8B-Instruct/Gemma2-2B-Instruct	Qwen2.5-Math-7B	DeepSeek-V3

We compare the open-source models’ choices of key technical components, accessibility, target area, and parameter scales, respectively.

Generally, these open-source frameworks not only demonstrate distinct implementation strategies for reinforced reasoning but also play an essential role in improving the understanding of OpenAI’s o1 model. Together, they expand the range of techniques available to the open-source community, advancing the collective goal of developing sophisticated, transparent, and adaptable reasoning models that bring proprietary-level capabilities to publicly accessible systems.

## Other test-time enhancing techniques

**Figure 6:**
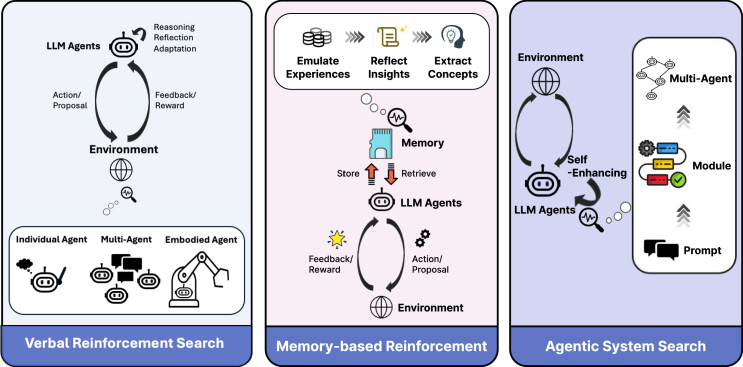
Typical training-free test-time enhancing methods: Verbal reinforcement search, memory-based reinforcement, and agentic system search

In addition to the PRM-guided search, there are numerous other techniques devised to enhance LLMs’ reasoning capabilities with more test-time compute. These techniques refine reasoning result dynamically without modifying the model itself. Approaches such as verbal reinforcement search, memory-based reinforcement, and agentic system search, depicted in Figure 6, demonstrate that substantial reasoning improvements can be achieved with off-the-shelf LLMs alone. A selection of representative works exploring these methods is summarized in Table 6. While these methods do not leverage PRM, they offer a foundation for future research to explore hybrid models for further advancing reasoning capabilities.

**Table 6:** A list of representative works of training-free test-time reinforcing

Method	Category	Representative literature
Verbal reinforcement search	individual agent	Romera et al.^123^; Shojaee et al.^124^; Mysocki et al.^125^; Ma et al.^126^
	multi-agent	Chen et al.^127^; Zhou et al.^128^; Le et al.^129^; Yu et al.^130^
	embodied agent	Boiko et al.^131^
Memory-based reinforcement	experiential learning	Zhang et al.^132^; Gao et al.^133^; Qian et al.^134^
	reflective learning	Shinn et al.^135^; Sun et al.^136^^,^^137^
	concept learning	Zhang et al.^138^; Gao et al.^139^; Guan et al.^140^
Agentic system search	prompt level	Madaan et al.^141^; Fernando et al.^142^; Yang et al.^143^
	module level	Shang et al.^144^; Zhang et al.^145^
	agent level	Huot et al.^146^; Zhuge et al.^147^

### Verbal reinforcement search

Verbal reinforcement search (VRS) leverages the pretrained reasoning and semantic capabilities of LLMs to explore and optimize solution spaces. Unlike traditional reinforcement learning or training-intensive approaches, VRS operates purely through test-time inference, using iterative feedback loops to refine solutions without requiring additional training. By drawing on the semantic knowledge encoded in LLMs and their ability to follow complex instructions, VRS provides a versatile approach for navigating diverse problem spaces. This inference-driven framework finds application across individual agents, multi-agent systems, and embodied agents, supporting a wide range of tasks, including programmatic optimization, collaborative decision-making, and interactions in real-world settings. This section analyzes VRS through these three key aspects, delving into the methodologies and unique insights presented within each category.

In *individual agent settings*, VRS relies on iterative reasoning and feedback mechanisms to refine solutions within structured problem spaces. This approach is well-suited for tasks like mathematical optimization, symbolic reasoning, and hypothesis-driven discovery, where systematic refinement significantly improves problem-solving outcomes. Research on mathematical discovery illustrates how VRS reshapes the problem-solving process into a dynamic iterative cycle. For example, studies on combinatorial problems, including the cap set and online bin packing, highlight how programmatic solutions evolve through feedback-driven evaluation.^123^ Similarly, symbolic regression research treats equations as dynamic constructs, iteratively generating, evaluating, and optimizing mathematical expressions.^124^ These approaches show how VRS navigates constrained spaces, surpassing traditional optimization techniques in efficiency and accuracy. In scientific discovery, VRS has shown its utility in integrating reasoning with empirical data and simulations. Researchers have developed systems for biomedical hypothesis refinement by synthesizing diverse data sources. For instance, applications in oncology use iterative synthesis to address the complexity of multi-scale data.^125^ In physical sciences, VRS is used to refine hypotheses through simulation feedback, advancing fields like molecular design and the discovery of physical laws.^126^ These findings emphasize the role of VRS in connecting abstract reasoning with real-world validation, supporting tasks that are both data intensive and hypothesis driven. Reflective processes in heuristic optimization further showcase the flexibility of VRS. For example, researchers have explored the iterative generation and evaluation of strategies for solving combinatorial problems.^148^ This approach focuses on creating adaptive hyper-heuristics that generalize effectively across different domains by continuously refining solutions through feedback cycles. Overall, VRS applies iterative reasoning and feedback to connect abstract problem-solving with real-world applications, addressing challenges in mathematics, science, and optimization with precision and adaptability.

In *multi-agent systems*, VRS facilitates collaboration between LLM agents through natural language communication. These systems leverage shared reasoning and iterative refinement to tackle complex solution spaces, allowing agents to exchange insights and achieve common goals. Meta-structure discovery in heterogeneous information networks (HINs) exemplifies how VRS is applied in multi-agent contexts. Recent research has combined LLM reasoning with evolutionary optimization to refine meta-structures, enhancing their explainability and predictive accuracy.^127^ Similarly, in socioeconomic prediction, multi-agent systems integrate knowledge graphs and meta-path reasoning to extract cross-task insights for applications like population estimation and economic activity prediction. This approach facilitates collaboration between LLM agents and improves performance in multi-task environments.^128^ Causal discovery also benefits from multi-agent frameworks enabled by VRS. For example, systems using LLMs as reasoning agents collaboratively debate and propose causal relationships. By incorporating statistical methods and natural language interactions, these frameworks generate accurate causal graphs while addressing ambiguities in causal relationships.^129^ In financial decision-making, VRS enhances hierarchical collaboration. The FINCON framework employs a manager-analyst system to refine financial strategies using conceptual verbal reinforcement. By minimizing redundant communication and improving strategy refinement, FINCON demonstrates the utility of VRS in optimizing financial decision-making processes.^130^ With iterative refinement and shared reasoning, VRS supports multi-agent systems in tackling complex tasks such as meta-structure refinement, socioeconomic prediction, and financial decision-making.

In *embodied agent settings*, VRS is used to address real-world tasks by integrating reasoning with physical interactions, supporting activities such as experimental planning and execution in laboratory settings. These systems extend VRS into dynamic environments, combining semantic reasoning with practical experimentation. For example, autonomous chemical research has demonstrated the use of LLM-powered systems to independently design, execute, and refine experiments.^131^ These agents integrate tools such as robotic liquid handlers, spectrometry devices, and web-based research modules to perform tasks like reaction optimization and compound synthesis. One application involves optimizing palladium-catalyzed cross-coupling reactions, where the system uses natural language prompts to determine conditions, calculate stoichiometries, and autonomously execute experiments. When faced with errors, such as incorrect module calls, the system revises its approach by referencing documentation and iterating on the task. This iterative process demonstrates how VRS supports adaptability and precision in experimental workflows. By combining reasoning and real-time feedback, embodied agents illustrate the capability of VRS to refine and optimize complex processes in dynamic environments. These systems reduce human intervention while accelerating scientific discovery, making them a valuable tool for real-world experimentation and innovation.

In general, previous studies have showcased the adaptability and effectiveness of VRS across individual agents, multi-agent systems, and embodied agents. Leveraging the semantic reasoning and iterative feedback capabilities of LLMs, VRS tackles a wide range of tasks without the need for additional training. From structured optimization in mathematical and scientific contexts to collaborative exploration in multi-agent frameworks and dynamic experimentation in real-world applications, VRS provides a unified approach to problem-solving. VRS as a versatile framework, capable of addressing complex challenges across both computational and physical domains while driving advances in diverse fields.

### Memory-based reinforcement

When applied to open-ended tasks such as creative writing, complex logical reasoning, and open-world gaming, the solution space tends to expand dramatically, often becoming unbounded or ill-defined. These tasks typically require continuous interaction with the environment to acquire relevant information, making simple solution space searches inefficient. To address these challenges, some studies incorporate an external memory module for LLM agents. This module stores information such as observations and successful and failed actions from past trials. Agents explore their environments iteratively, using memory as a foundation for verbal reinforcement learning. Through this process, they summarize experience, extract interpretable high-level insights of the solution space, and refine their actions in subsequent trials, thereby improving inference performance. These studies not only focus on exploring the external solution space but also emphasize the intrinsic ability of LLM agents to develop an understanding of the solution space from memory. As the agents accumulate memory through environmental exploration, their capabilities are progressively reinforced and generalized to unseen tasks. Specifically, we classify the studies in this area into the following three categories.

#### Experiential learning

Methods in this category encourage LLM agents to simply emulate favorable experiences stored in memory while avoiding unfavorable ones. REMEMBERER^132^ introduces a semi-parametric RL-LLM agent that records past observation-action pairs in memory and uses a traditional off-policy Q-learning algorithm to dynamically maintain and update the Q-value (expected future reward) of each observation-action pair. When faced with a new task, the agent retrieves relevant actions with the highest and lowest Q-values from memory, incorporating these as encouraged and discouraged examples in the prompt. Memory sharing^133^ leverages concepts from multi-agent reinforcement learning to enhance learning efficiency. Multiple agents execute tasks concurrently in a shared environment and contribute high-quality prompt-answer pairs to a collective memory pool. Each agent can retrieve the most relevant examples from this pool to facilitate few-shot learning. Similarly, experiential co-learning^134^ employs a multi-agent framework in which instructor and assistant agents alternately provide instructions and solutions during multi-step code generation. This dynamic exchange helps extract shortcuts to reduce redundancy and prevent repetitive mistakes. When encountering new tasks, these agents retrieve relevant memories alternately to improve in-context learning.

#### Reflective learning

Although using memory as few-shot exemplars is straightforwardly effective, this approach does not fully exploit the semantic comprehension capabilities of LLMs. Some studies argue that LLM agents should reflect directly on the successes and failures stored in memory to summarize underlying causes explicitly, adopting these insights as guidelines. Reflexion^135^ is a pioneering effort in this area, reflecting on the reasons behind success or failure semantically based on task feedback signals. It integrates reflective text and past trajectories into prompts to enhance decision-making in subsequent trials. ExpeL^137^ combines imitation and reflection by retrieving the most relevant successful experiences from memory, summarizing patterns of successful trajectories, and identifying insights from comparisons of success-failure pairs. RAHL,^136^ inspired by hierarchical reinforcement learning, organizes memory into goal modules and sub-task modules, enabling reflection and experience summarization at different levels. For new tasks, it retrieves relevant experience to formulate high-level goals and low-level sub-tasks separately.

#### Concept learning

Explicit reflection significantly enhances the inference capabilities of LLMs. Building on this, some studies aim to enable LLM agents to develop generalized “concepts” that transcend specific tasks, facilitating a broader understanding of the environment and tasks. This generalization helps agents internalize cognitive abilities from memory and evolve continuously as memory grows. Agent-Pro,^138^ for example, enables agents to establish beliefs about themselves and their environments in card-based gaming. Instead of reflecting on individual actions, it evaluates the rationality and consistency of these beliefs, iteratively refining strategies. Similarly, Richelieu^140^ equips agents with an understanding of the environment in military strategy games. It retrieves the most relevant states from memory to formulate plans and assess feasibility. By employing self-play, it collects experience autonomously, assuming the roles of all players to advance its knowledge. Self-Evolving GPT,^139^ inspired by human memory mechanisms, designs a memory-based autonomous learning framework for LLMs. It categorizes tasks to determine relevant memory retrievals and identifies differences between stored memories and the current task to extract shared general experience. Additionally, it generates unseen tasks for practice, consolidating its knowledge based on memory retrieval outcomes.

### Agentic system search

The design of agentic systems plays a crucial role in harnessing the power of LLMs for many downstream tasks. An important branch of test-time enhancing techniques is to leverage LLMs to search the agentic systems. Studies in this area can be classified into three levels of search: prompt level, module level, and agent level. Please note that this approach does not aim to directly search the solution space; rather, it leverages the empirical data to optimize the agentic system itself, which is similar to a meta-learning problem. We summarize the related works in this area as follows.

#### Prompt level

The process of “verifying and correcting” improves the prompts by iteratively integrating useful feedback experience. The verification signal can come from external feedback,^149^ LLM’s self-evaluation,^141^ and other sources. On the other hand, prompts themselves are also worth searching and optimizing. Automated prompt engineering, such as evolutionary prompt optimization^142^ and meta prompt iterations,^143^ can achieve better results than manual prompts, but it also introduces more token consumption.

#### Module level

Agentsquare^144^ proposes to use LLMs to search the modular design of agentic systems, where the modules are essentially blocks of prompts that have specific functions of planning, reasoning, tool use, and memory. The basic units of these agentic modules have standard IO interface that allows them to collaborate well with each other. The advantage of module level search is it allows new agents to easily reused the classic agent design, such as CoT and ToT, through recombination of modules. Besides, Aflow^145^ connects the different calling nodes of LLM through edges represented by code. In addition to the search method, it is necessary to evaluate the performance of the searched agents. The function used to evaluate the performance of agents can also be driven by LLMs to improve search efficiency while closely matching their actual performance.

#### Agent level

ADAS proposes to leverage LLMs to search the entire agentic systems defined in Python code space.^150^ Besides, multi-agent systems make decisions and achieve goals in a shared environment. In multi-agent level search, key aspects include agent creation, environmental perception, action, interaction, and system evolution. The search for multi-agent systems has achieved good results in downstream tasks such as long story creation.^146^ The unified search and optimization mechanism for multi-agent systems is currently being explored. GPTSwarm^147^ enhances the collaborative capability of agents through graph optimization.

Agentic system search provides agents with the ability to self-improve, enabling them to optimize themselves to enhance their reasoning abilities without the need to make changes to the LLM structure. The above three levels of search have vast search spaces. The common challenge faced by these three search levels is to improve search efficiency, reduce search costs, and ensure automation while ensuring search rationality.

### Summary

The test-time enhancing techniques review in this section currently are not incorporated in the implementations of large reasoning models. However, they have huge potential to further boost the reasoning capacities of LLMs through more comprehensive test-time “thinking,” facilitating LLMs to strategically reason across the solution space, leverage past experiences and dynamically optimize agentic workflows. Therefore, training LLMs to master these test-time techniques represents a promising future research direction, with the potential to elevate LLMs from “reasoners” to fully functional “agents.”

## Evaluation benchmarks

**Figure 7:**
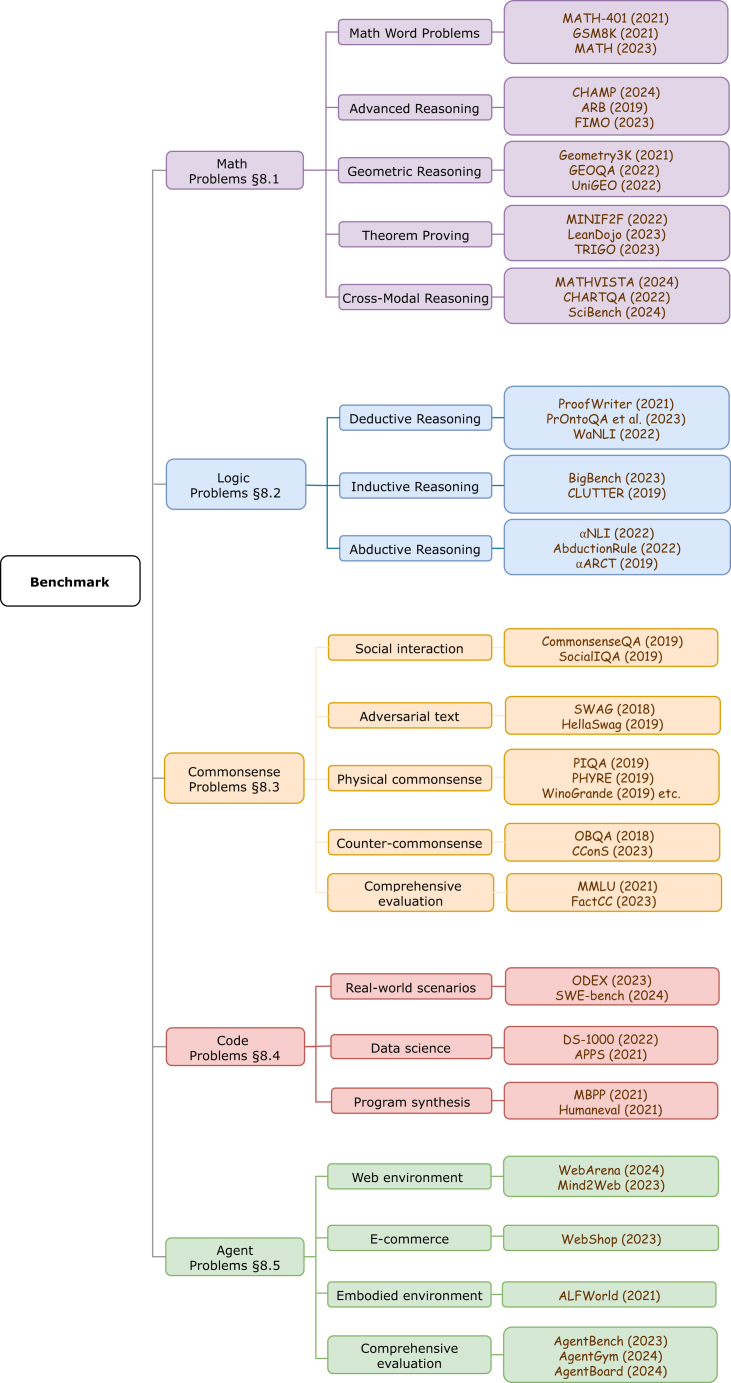
A taxonomy for LLM reasoning benchmarks

Designing a robust benchmark is important to document the improvement in LLM’s capabilities. It also plays a crucial role in selecting the promising research direction for further advance. In this section, we systemically review the popular benchmarks for LLM reasoning, which are summarized with the taxonomy in Figure 7.

### Math problems

Mathematical reasoning has become a crucial testbed for evaluating LLMs’ reasoning capabilities. The landscape of mathematical reasoning benchmarks spans from elementary arithmetic to advanced university-level mathematics, providing systematic ways to assess different aspects of mathematical understanding and problem-solving abilities.

In the realm of *mathematical word problems* (MWPs), benchmarks progress from fundamental arithmetic operations to increasingly complex problem-solving scenarios. At the basic level, datasets like MATH-401^151^ evaluate pure arithmetic capabilities through 401 carefully structured expressions, while MultiArith^152^ and AddSub^153^ assess the ability to translate simple word problems into mathematical operations (such as addition or subtraction). Moving to elementary and high school levels, comprehensive datasets such as GSM8K^154^ and MATH^155^ present more sophisticated multi-step reasoning challenges, with GSM8K offering 8.5k grade-school problems and MATH providing 12.5k problems across various mathematical domains with graduated difficulty levels.

The evaluation of *advanced mathematical capabilities* is primarily conducted through competition and specialized test datasets. Collections like CHAMP^156^ and ARB^157^ present competition-level problems that require sophisticated problem-solving strategies, while MATHQA^157^ incorporates standardized test questions from GRE and GMAT examinations. At the highest level, datasets such as FIMO^158^ challenge models with International Mathematical Olympiad problems, testing the limits of automated mathematical reasoning.

*Geometric reasoning* represents a distinct category requiring spatial understanding and formal mathematical proofs. Datasets like Geometry3K^159^ and GEOQA^160^ provide specialized geometric problems, while UniGEO^161^ offers a unified framework for geometric reasoning tasks focusing on calculation and proving. These benchmarks are particularly valuable in assessing models’ abilities to connect visual and mathematical reasoning.

The field of *theorem proving* and formal mathematics has evolved to include rigorous evaluation frameworks. MINIF2F^162^ and LeanDojo^163^ focus on formal mathematical proofs related to Lean Theorem, while THEOREMQA-MATH^164^ examines understanding of mathematical theorems. Specialized datasets like TRIGO^165^ and PISA^166^ address specific areas of mathematical reasoning, such as trigonometry and formal proof systems.

Lastly, *cross-modal mathematical reasoning* has emerged as a crucial area, reflecting the diverse ways mathematical problems are presented in real-world scenarios. MATHVISTA^167^ and CHARTQA^168^ evaluate visual mathematical reasoning through diagrams and charts, while TABMWP^169^ and MultiHiertt^170^ assess the ability to reason with tabular and textual data. SciBench^171^ bridges the gap between pure mathematics and scientific applications, testing mathematical reasoning in broader scientific contexts.

### Logical problems

Building upon mathematical reasoning capabilities, the ability to engage in systematic logical reasoning stands as another fundamental criterion for evaluating LLM’s cognitive abilities. While mathematical reasoning focuses on quantitative operations and formal proofs, logical reasoning encompasses the broader capacity to draw valid conclusions, recognize patterns, and generate rational explanations across diverse contexts. According to Luo et al.,^172^ logical reasoning can be classified into three main types: deductive, inductive, and abductive reasoning. Each type represents a distinct cognitive process essential for comprehensive logical analysis while maintaining interconnections in cognitive assessment.

*Deductive reasoning*, also known as premise-based reasoning, involves deriving specific conclusions from general principles with absolute certainty. For example, given a set of rules about relationships between entities, a model must determine what specific relationships must be true. ProofWriter^173^ exemplifies this category, requiring models to construct explicit logical derivations from given premises. Other benchmarks, such as FOLIO^174^ and PrOntoQA^175^ evaluate first-order logic reasoning in natural contexts; WaNLI^176^ has introduced increasingly sophisticated evaluation criteria with 107,885 examples.

*Inductive reasoning* emphasizes pattern recognition and generalization from specific observations to broader principles.^177^ This involves identifying underlying regularities and extending them to new situations, dealing with probability rather than certainty. BigBench^178^ with numerous specialized components that examine advanced pattern inference capabilities. Also, the CLUTTR^179^ benchmark series evaluates this capability through relationship patterns of varying complexity.

*Abductive reasoning*, also termed explanatory reasoning, refers to the process of forming the most likely explanation for a set of observations or facts, even though the conclusion is not guaranteed to be certain.^180^ This type of reasoning tests how models handle scenarios with incomplete information by generating reasonable explanations. The α NLI^181^ benchmark implements this through narrative completion tasks, where models must select the most likely explanation for given situations. The AbductionRule^182^ series offers structured evaluation frameworks across different domains, with specific variants for animal-related and person-related reasoning scenarios. α ARCT^183^ specifically examines the ability to select and justify plausible explanations and argument comprehension.

### Common-sense problems

Common-sense reasoning remains a significant challenge in NLP, as it aims to evaluate LLMs’ ability to understand and apply everyday commonsense knowledge. There are various benchmarks targeting different dimensions of commonsense reasoning tasks. For instance, CommonsenseQA^184^ requires models to answer reasoning questions grounded in commonsense knowledge bases.

SocialIQA^185^ focus on *social interaction* common-sense reasoning, which revolves around causal reasoning in social scenarios. In contrast, datasets like SWAG^186^ and HellaSwag^187^ introduce *adversarial text* reasoning tasks, where models must predict the most plausible continuation of events based on contextual clues, thereby increasing task complexity. For *physical common-sense* reasoning, benchmarks such as PIQA^188^ and PHYRE^189^ concentrate on evaluating models’ understanding of everyday physical tasks and interactive reasoning scenarios. PIQA primarily uses question-answering tasks, while PHYRE emphasizes interactive physical simulations. Similarly, WinoGrande^190^ builds upon the Winograd Schema Challenge by introducing a larger-scale dataset and more complex disambiguation tasks to test semantic understanding and coreference resolution capabilities.

Other works, such as OBQA^191^ and CConS,^192^ explore model performance in *counter-commonsense* contexts, highlighting the challenges faced by current models in implicit reasoning and background knowledge utilization. More recently, *comprehensive benchmarks* like MMLU^193^ and critical studies such as FactCC^194^ have further analyzed LLM’s commonsense reasoning and factual reasoning. These benchmarks offer valuable perspectives on the generalization abilities of language models and serve as valuable tools for evaluating and improving their performance across diverse commonsense reasoning tasks.

### Coding problems

The development of code generation benchmarks has been instrumental in evaluating the reasoning capabilities of LLMs in programming tasks. These benchmarks assess models’ proficiency in generating accurate, efficient, and reliable code across various domains. For example, ODEX^195^ introduces an execution-driven evaluation framework for open-domain code generation, emphasizing the importance of running generated code to verify its correctness and functionality.

As for the *real-world scenarios*, SWE-bench^196^ focuses on real GitHub issues, challenging models to resolve practical software engineering problems. In the realm of *data science*, DS-1000^197^ presents a benchmark featuring authentic and dependable data science code generation tasks, enabling the assessment of models’ abilities to handle complex data manipulations and analyses. Besides, the APPS benchmark^193^ measures coding challenge competence by evaluating models on a diverse set of programming problems, reflecting the challenges encountered in competitive programming and technical interviews.

MBPP^198^ focuses on *program synthesis* problems, assessing models’ abilities to generate correct and efficient code based on given specifications, thereby contributing to the understanding of LLMs’ capabilities in automated code generation. The HumanEval^199^ evaluates LLMs trained on code by providing a set of Python programming problems, each provided with a function definition and accompanying documentation, requiring models to generate correct and functional code solutions.

### Agent problems

The emergence of agent-based benchmarks has revolutionized our ability to assess LLMs as independent agents within interactive environments. These sophisticated evaluation frameworks assess crucial capabilities including decision-making, reasoning, and environmental interaction across diverse scenarios.

WebArena^200^ provides a practical *web environment* for building and testing autonomous agents, enabling the evaluation of LLMs’ web navigation and interaction skills. Similarly, Mind2Web^9^ aims to develop generalist agents capable of operating across diverse web tasks, emphasizing adaptability in dynamic online environments.

In *e-commerce* settings, WebShop^201^ introduces a platform for scalable real-world web interaction, focusing on grounded language agents that can perform tasks such as online shopping, thereby testing models’ practical application abilities. To bridge textual and embodied environments, ALFWorld^202^ aligns text-based inputs with interactive learning scenarios, facilitating the assessment of models’ abilities to transfer knowledge between different modalities.

*Comprehensive evaluation* frameworks like AgentBench^203^ and AgentGym^204^ have been developed to systematically assess LLMs functioning as agents. AgentBench includes diverse environments to assess reasoning and decision-making skills, while AgentGym focuses on evolving LLM-based agents across diverse settings, emphasizing adaptability and learning efficiency. Furthermore, AgentBoard^205^ offers an analytical platform for evaluating multi-turn LLM agents, providing insights into their performance over extended interactions and highlighting areas for improvement in sustained reasoning tasks.

### Performance trends and future directions

**Figure 8:**
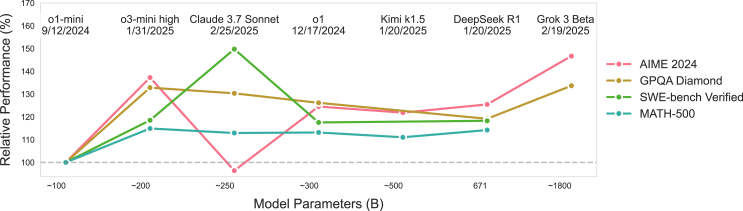
Performance trend of the recently released large reasoning models

To contextualize the progression toward large reasoning models, we analyze performance trends on several challenging benchmarks, including AIME 2024, GPQA Diamond, SWE-bench Verified, and MATH-500, as shown in Figure 8. Our analysis focuses on recently released large-scale models (with over 100 billion parameters) developed by leading organizations in the field. Performance data are drawn from official model reports, and for closed-source models, parameter sizes are estimated based on community consensus. To highlight relative improvements across diverse benchmarks, we normalize all reported scores as performance gains relative to OpenAI’s o1-mini, the first publicly acknowledged reasoning model. The results indicate that recent models exhibit substantial improvements over o1-mini. Interestingly, models ranging from 200B to 671B parameters demonstrate comparable performance, suggesting that recent advances in reasoning capabilities are increasingly driven not by model scaling alone, but by innovations in post-training techniques and test-time inference strategies. Notably, Grok 3 Beta shows further gains beyond this plateau, and Claude 3.7 Sonnet exhibits a particular strength in coding tasks on SWE-bench Verified.

As reasoning capabilities of LLMs continue to improve, many popular benchmarks are increasingly becoming saturated or outdated. In mathematical reasoning, datasets like GSM8K^154^ and MATH^155^ are centered on grade-school and high-school problems, on which advanced models now achieve near-perfect accuracy—limiting their ability to discriminate between stronger models. In code generation, benchmarks such as HumanEval^199^ and MBPP^198^ include tasks solvable without advanced reasoning, reducing their evaluative value. Similarly, agent-based environments like WebArena^200^ and WebShop^201^ often rely on basic execution skills rather than complex decision-making or reasoning. Therefore, there is an urgent need for the development of more complex, dynamic, and interdisciplinary evaluation benchmarks that better capture real-world reasoning challenges and can meaningfully guide future research.

## Discussion

### Inspirations from the recent advances

#### Scaling law of post-training phases

The inspiration from OpenAI’s o1 series leads to a new understanding of the pretraining/post-training/inference stages. Particularly, it involves the introduction of self-play reinforcement learning and the process reward learning of high-quality chain-of-thought labeled data during the post-training stage. Further, it extends to the scaling law in the post-training stage, which provides inspiration for the difficulties in the further development of the scaling law in the training stage. As we know, the scaling law in the pretraining and training stages has led to the success of popular LLMs, with the huge investment of training data and computation resources. However, it now reaches the bottleneck, and thus, the scaling law of post-training phases may be the driving force for the next period of development of LLMs. Furthermore, LLM agents^206^ have also shown great potential with carefully designed workflow even if the reasoning abilities have not been reinforced. Therefore, it is still an open question whether there will also be a similar scaling law regarding resource consumption and performance in LLM agents, which could be the potential to further enhance LLM in real-world applications. Lastly, there may be a relationship between the currently exhibited test-time scaling law and the model’s ability to follow instructions; that is, it must have a sufficiently strong instruction following ability to demonstrate test-time scaling laws. For example, the success of verbal reinforcement search techniques require the LLMs to have the basic ability to follow instructions. Thus, if the LLMs cannot accurately follow instructions, the complicated post-training techniques might not work properly.

#### Generating high-quality data through searching

Both the technical ideas of OpenAI’s o1 series disclosed by its core technical personnel and the open-source works attempting to reproduce OpenAI’s o1 currently regard the generation of high-quality data (including CoT data) as the key point, although different approaches such as Monte Carlo tree search, LLM generation, and others have been adopted. That is, the development of large reasoning models reaches a stage where high-quality process reward data are more important than general pretraining data size. Similarly, as discussed above, it may inspire us to refer to these related approaches in LLM agents as well, first to conduct high-quality data generation and then enhance the learning of slow reasoning as well as the acquisition of capabilities.

### Slow-thinking and reasoning

Even if the breakthrough of OpenAI’s o1 series at the engineering level remains unknown, theoretically and technically, its breakthrough currently seems to mainly lie in the post-training learning of slow-thinking data. Also, the human cognitive science of “system 1 + system 2” has been repeatedly mentioned, but the ideas on how to implement it based on large models have been constantly updated, mainly still staying at the stage of drawing on the concept of slow thinking. That is, the mechanism named “system 1 + system 2” of human brains has guided the design of LLMs, but the guidance is still very limited. In other words, the imitation of the human brain is only about system-level design rather than very detailed techniques. The complex mechanisms of human slow thinking and their benefits still show high potential to support the next-level reasoning abilities of LLMs. To accomplish it, the domain knowledge of slow thinking should be used in the related designs such as reasoning data generation, reward functions, learning process, etc.

There has been no truly significant and representative work on the theoretical analysis of slow-thinking of LLMs up to now. The generative artificial intelligence is so mysterious that understanding LLMs also requires some tricks or special techniques such as new metrics for understanding hallucination in LLMs.^207^ To understand the slow-reasoning abilities, we may need to also step into the theoretical analysis. Taking the two different versions, OpenAI’s o1-preview and o1-mini, as examples, the main difference lies in the cost and depth of thinking in the CoT inference stage, yet they show significant differences in tasks such as text generation, code generation, and mathematical problem-solving. The special characteristics of reasoning shown by LLMs also inspire us to design task-adaptive usage and applications. Specifically, it may support more interesting insights to link the reasoning mechanism and the performance in different tasks.

### Downstream applications and open problems

As this paper highlights, the pace of progress in reasoning enhancement technologies is remarkable. The capabilities of these technologies now extend beyond standard benchmarks to increasingly complex, general-purpose tasks in downstream applications. For example, the FunSearch framework^123^ has shown the general ability for tasks that are challenging to solve but straightforward to verify. A host of tasks with similar characteristics likely exist in diverse domains such as urban planning and logistics scheduling. Even in the most demanding frontiers of scientific research requiring profound originality, vast opportunities for exploration are emerging. DeepMind’s recent AlphaEvolve^208^ serves as a prime example; by employing a novel evolutionary algorithm to navigate a vast possibility space, it has achieved historic breakthroughs on intractable mathematical challenges like the kissing number^208^ and the sum and difference of sets problem,^209^ further illustrating the boundless potential of search-based reasoning.

This trend raises an intriguing question: might there be a complementary class of problems in current research characterized by high verification difficulty but a comparatively straightforward reasoning process, such as modern mathematical proofs, medical diagnosis assistance, legal reasoning, code verification, peer review in scientific research, or security vulnerability detection? In such cases, it may be possible to evaluate solution quality by combining LLMs with external evaluation tools. These scored outputs could also be repurposed as training data to develop more advanced reward models. Recent studies have started exploring the use of LLMs as evaluators. Fusion-Eval,^210^ for example, is an LLM-based framework that integrates multiple auxiliary evaluators to significantly improve correlation with human judgment. Leveraging in-context learning and role-playing capabilities, LLMs themselves can also serve as specialized evaluators that can be easily generalized to new evaluation scenarios.^211^ However, it is important to note that when serving as evaluators, the inherent limitations of LLMs themselves must be carefully addressed, such as their susceptibility to prompt-template influence, which can introduce inconsistency in evaluation results.^212^ The vast corpora used for their training may also cause them to inherit and propagate implicit biases, thereby compromising the fairness and reliability of their assessments.^213^

In light of these challenges, exploring collaborative reasoning between humans and AI presents a promising path forward. On one hand, reasoning models offer distinct advantages: they can process information at a massive scale, exhibit high degrees of scalability and interpretability, and possess the capacity to uncover patterns and correlations that may elude human perception. On the other hand, human faculties such as experience, intuition, and wisdom represent capabilities that current models cannot replicate. Humans can grasp the fundamental insights from AI-generated findings and leverage this understanding for higher-order cognitive tasks like abstraction, generalization, and theoretical synthesis.^208^ A synergistic partnership between these two holds the potential to unlock new paradigms for solving complex reasoning problems.

## Conclusion

In conclusion, the evolution of LLMs has significantly advanced their humanlike reasoning capabilities, marking a pivotal shift from simple token generation to complex cognitive processing. The introduction of “thought” as a sequence of intermediate steps has been a critical innovation, laying the groundwork for more sophisticated large reasoning models. The synergistic combination of automated data construction, train-time scaling through reinforcement learning, and test-time scaling via guided search algorithms has proven essential in unlocking these advanced capabilities. As exemplified by OpenAI’s o1 series and various open-source efforts, these models are increasingly able to tackle complex problems in domains like mathematics and science by decomposing problems and verifying solutions. Looking forward, the development of more powerful reasoning models will likely depend on overcoming key challenges in data generation and learning algorithms. A promising frontier lies in exploring human-AI collaborative reasoning, where the massive-scale processing of models complements human intuition and experience. Furthermore, enhancing LLMs’ ability to perform “slow thinking” and master test-time search and reflection techniques represents a significant research direction, with the potential to elevate them from mere “reasoners” to fully functional and autonomous “agents.” This transformation entails not only advanced reasoning but also capabilities such as tool use for interacting with external systems, strategic planning over long horizons, dynamic decision-making under uncertainty, and multi-modal perception that integrates diverse data sources. Together, these attributes enable agents to operate flexibly in complex, real-world environments with minimal human intervention. This ongoing progress not only promises to reshape our understanding of language and cognition but also heralds a new era of AI-driven solutions to complex real-world problems.

## Acknowledgments

F.X. received support from the 10.13039/501100001809National Natural Science Foundation of China (#23IAA02114 and #62472241).

## Declaration of interests

The authors declare no competing interests.
